# Circulating ECM proteins decorin and alpha-L-iduronidase differentiate ATTRwt-CM from ATTRwt-negative HFpEF/HFmrEF

**DOI:** 10.1093/cvr/cvae189

**Published:** 2024-09-17

**Authors:** Alwin Tubben, George Markousis-Mavrogenis, Laura M G Meems, Bart J van Essen, Lukas Baumhove, Milou Berends, Hendrea S A Tingen, Johan Bijzet, Bouke P C Hazenberg, Adriaan A Voors, Dirk J van Veldhuisen, Riemer H J A Slart, Hans L A Nienhuis, Peter van der Meer

**Affiliations:** Department of Cardiology, University Medical Centre Groningen, 9713GZ Groningen, The Netherlands; Amyloidosis Centre of Expertise, 9713GZ Groningen, The Netherlands; Department of Cardiology, University Medical Centre Groningen, 9713GZ Groningen, The Netherlands; Department of Cardiology, University Medical Centre Groningen, 9713GZ Groningen, The Netherlands; Department of Cardiology, University Medical Centre Groningen, 9713GZ Groningen, The Netherlands; Department of Cardiology, University Medical Centre Groningen, 9713GZ Groningen, The Netherlands; Amyloidosis Centre of Expertise, 9713GZ Groningen, The Netherlands; Department of Internal Medicine, University Medical Centre Groningen, 9713GZ Groningen, The Netherlands; Amyloidosis Centre of Expertise, 9713GZ Groningen, The Netherlands; Department of Nuclear Medicine and Molecular Imaging, University Medical Centre Groningen, 9713GZ Groningen, The Netherlands; Amyloidosis Centre of Expertise, 9713GZ Groningen, The Netherlands; Department of Laboratory Medicine, University Medical Centre Groningen, 9713GZ Groningen, The Netherlands; Amyloidosis Centre of Expertise, 9713GZ Groningen, The Netherlands; Department of Internal Medicine, University Medical Centre Groningen, 9713GZ Groningen, The Netherlands; Department of Cardiology, University Medical Centre Groningen, 9713GZ Groningen, The Netherlands; Department of Cardiology, University Medical Centre Groningen, 9713GZ Groningen, The Netherlands; Amyloidosis Centre of Expertise, 9713GZ Groningen, The Netherlands; Department of Nuclear Medicine and Molecular Imaging, University Medical Centre Groningen, 9713GZ Groningen, The Netherlands; Amyloidosis Centre of Expertise, 9713GZ Groningen, The Netherlands; Department of Internal Medicine, University Medical Centre Groningen, 9713GZ Groningen, The Netherlands; Department of Cardiology, University Medical Centre Groningen, 9713GZ Groningen, The Netherlands; Amyloidosis Centre of Expertise, 9713GZ Groningen, The Netherlands

**Keywords:** Cardiac amyloidosis, ATTR wild-type, HFpEF, HFmrEF, Biomarker, Pathophysiology

## Abstract

**Aims:**

Wild-type transthyretin cardiac amyloidosis (ATTRwt-CM) is an under-recognized aetiology of heart failure (HF), necessitating early detection for timely treatment. Our study aimed to differentiate patients with ATTRwt-CM from ATTRwt-negative HFpEF/HFmrEF patients by identifying and validating circulating protein biomarkers. In addition, we measured the same biomarkers in patients with cardiomyopathy due to light chain amyloidosis (AL)-CM to gain disease-specific insights.

**Methods and results:**

In this observational study, serum concentrations of 363 protein biomarkers were measured in a discovery cohort consisting of 73 ATTRwt-CM, 55 AL-CM, and 59 ATTRwt-negative HFpEF/HFmrEF patients, using multiplex proximity extension assays. Sparse partial least squares analyses showed overlapping ATTRwt-CM and AL-CM biomarker profiles with clear visual differentiation from ATTRwt-negative patients. Pathway analyses with g:Profiler revealed significantly up-regulated proteoglycans (PG) and cell adhesion pathways in both ATTRwt-CM and AL-CM. Penalized regression analysis revealed that the proteoglycan decorin (DCN), lysosomal hydrolase alpha-L-iduronidase (IDUA) and glycosyl hydrolase galactosidase β-1 (GLB-1) most effectively distinguished ATTRwt-CM from ATTRwt-negative patients (*R*^2^ = 0.71). In a prospective validation cohort of 35 ATTRwt-CM patients and 25 ATTRwt-negative patients, DCN and IDUA significantly predicted ATTRwt-CM in the initial analysis (DCN: OR 3.3, IDUA: OR 0.4). While DCN remained significant after correcting for echocardiographic parameters, IDUA did not. DCN showed moderate discriminative ability (AUC, 0.74; 95% CI, 0.61–0.87; sensitivity, 0.91; specificity, 0.52) as did IDUA (AUC, 0.78; 95% CI, 0.65–0.91; sensitivity, 0.91; specificity, 0.61). A model combining clinical factors (AUC 0.92) outperformed DCN but not IDUA, a combination of the biomarkers was not significantly better. Neither DCN nor IDUA correlated with established disease markers.

**Conclusion:**

ATTRwt-CM has a distinctly different biomarker profile compared with HFpEF/HFmrEF, while ATTRwt-CM patients share a similar biomarker profile with AL-CM patients characterized by up-regulation of proteoglycans and cell-adhesion pathways. The biomarkers DCN and IDUA show the potential to serve as an initial screening tool for ATTTRwt-CM. Further research is needed to determine the clinical usefulness of these and other extracellular matrix components in identifying ATTRwt-CM.

## Graphical Abstract

**Graphical Abstract:**
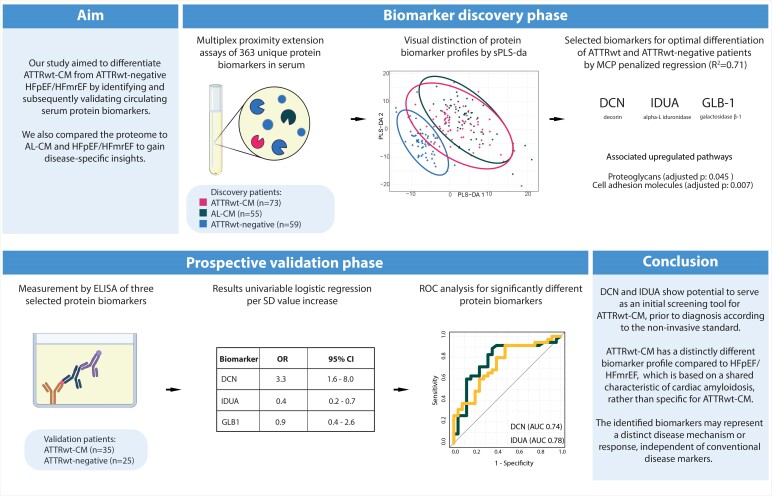

**Time of primary review: 31 days**

## 1. Introduction

Wild-type transthyretin cardiac amyloidosis (ATTRwt-CM) is an under-recognized aetiology of heart failure (HF).^1^ This type of infiltrative cardiomyopathy results from the extracellular myocardial deposition of misfolded transthyretin proteins, leading to HF, arrhythmias, cardiac conduction abnormalities and thromboembolism.^2^

Invasive and non-invasive criteria can accurately diagnose ATTRwt-CM.^1^ Yet, the recognition and differentiation between ATTRwt-CM and other common aetiologies of HF remain challenging due to overlapping clinical features.^1,3^ Diagnosing ATTRwt-CM at an early stage is imperative, because the only currently approved treatment for ATTRwt-CM is most beneficial in the early stages of HF and no curative treatments are available at this moment.^4^ Recent advancements in the understanding of ATTRwt-CM have led to the identification of specific populations that are more likely to have ATTRwt-CM, such as patients with HF with preserved or mildly reduced ejection fraction (HFpEF/HFmrEF) and left ventricular hypertrophy.^5–11^ Furthermore, the increased awareness of ATTRwt-CM, along with improvements in diagnostic criteria, screening, and monitoring methods, has coincided with a rise in the incidence of ATTRwt-CM in recent years.^1,12–16^ Still, diagnostic delay remains a significant hurdle to prompt treatment initiation.^17^

Biomarkers could provide clinicians with a simple and cost-effective tool to systematically screen for patients with an increased risk of having ATTRwt-CM, especially in pre-selected populations with higher *a priori* risk.^18^ Therewith, biomarker-based screening could potentially minimize diagnostic delay and prompt the early use of specialist diagnostic and therapeutic modalities. NT-proBNP, troponin T, uric acid, transthyretin (TTR), and renal function are suitable serum biomarkers for risk stratification of survival in ATTRwt-CM, but they cannot differentiate ATTRwt-CM from other common cardiac diseases.^19,20^

Currently, there are no disease-specific serum biomarkers available for the screening or diagnosis of ATTRwt-CM. This study aimed to address this gap by identifying and validating circulating protein biomarkers that can aid in the screening for ATTRwt-CM within a HFpEF/HFmrEF population. We also performed broader protein profile analyses, including patients with light chain (AL) amyloidosis to elucidate whether the identified biomarkers and underlying pathophysiology were specific to ATTRwt-CM or exhibited a broader relevance for cardiac amyloidosis in general when compared to HFpEF/HFmrEF.

## 2. Methods

### 2.1 Study design

This is an observational case-control study with an initial biomarker discovery phase, in which serum biomarkers that optimally differentiated between ATTRwt-CM discovery patients and ATTRwt-negative discovery patients with HFpEF/HFmrEF were identified. The optimally differentiating biomarkers from the discovery cohort were validated using enzyme-linked immunosorbent assays (ELISAs) in a prospective validation cohort consisting of subsequently recruited ATTRwt-CM validation patients and ATTRwt-negative validation patients with HFpEF/HFmrEF.

To provide insights into whether the identified biomarkers in discovery analyses were specific to ATTRwt or related to cardiac amyloidosis in general, the discovery phase included patients with AL-CM alongside ATTRwt-CM and HFpEF/HFmrEF patients.

### 2.2 Study population

The patients with ATTRwt-CM in the discovery cohort, ATTRwt discovery patients from hereon, were newly diagnosed, consecutively included, treatment naïve patients with ATTRwt-CM who visited the Amyloidosis Centre of Expertise of the University Medical Centre Groningen (UMCG) between January 2010 and December 2018.

The patients with ATTRwt-CM in the prospective validation cohort, ATTRwt validation patients from hereon, were newly diagnosed, consecutively included, treatment naïve patients with ATTRwt-CM that visited the Amyloidosis Centre of Expertise UMCG from January 2020 to September 2022. ATTRwt-CM was diagnosed according to the European Society of Cardiology position paper on diagnosis and treatment of cardiac amyloidosis.^1^

For a comparative ATTRwt-CM negative cohort, for both the discovery and prospective validation cohort, ATTR-negative discovery and validation patients from hereon, patients from the previously conducted Amylo-VIP-HF study were included.^11,21^ In this study, ATTRwt-CM was ruled out in 97 patients by [^99m^Tc]Tc-hydroxy-diphosphonate (HDP) scintigraphy and single photon emotion tomography (SPECT) between January 2015 and December 2019. Of the ATTRwt-negative patients, 74% had a left ventricular ejection fraction (LVEF) ≥50% (i.e. HFpEF), and all other patients had an LVEF between 40 and 50% (i.e. HFmrEF). Further details on the concise study population, design, and results can be read elsewhere.^11^ Serum samples were available of the 84 ATTRwt-negative patients and were randomly divided to be in either part of the ATTRwt-negative discovery cohort or ATTRwt-negative prospective validation cohort. Patients with HF with reduced ejection fraction (HFrEF) were excluded based on the above-mentioned inclusion criteria, aligned with the aim of early identification, as ATTRwt-CM typically presents as HFpEF/HFmrEF in earlier stages.^22,23^

Treatment naïve patients with AL-CM who visited the Department of Internal Medicine of the UMCG were consecutively included in a biobank from July 2007 until October 2019 if the diagnosis according to the consensus guideline had been made.^24^ No AL-CM patient were included for validation analysis, as serum-free light chains and serum and urine immunofixation can differentiate AL-CM accurately from HFpEF/HFmrEF or ATTRwt-CM.

No patients with variant ATTR-CM (ATTRv-CM) were included. Unlike ATTRwt-CM and AL-CM, which may be discovered unexpectedly, ATTRv-CM typically arises from family history and is actively screened for within known at-risk families in our experience.

All procedures were in compliance with the Declaration of Helsinki. The study was approved by the institutional review board of the UMCG (registration number 2012/296). Written informed consent was obtained from all subjects.

### 2.3 Serum samples

Ten millilitre blood samples of all included ATTRwt and ATTRwt-negative patients were collected at the central lab of the UMCG. All collected blood samples were centrifuged for 10 min at 2000 g. The serum was stored in 500 µL vials at −20°C, prior to central storage at −80°C.

### 2.4 Statistical analyses

Statistical analyses were performed using IBM SPSS version 28.0 (SPSS Inc., Chicago, IL, USA), and R-studio version 4.3.2 (R Foundation for Statistical Computing, Vienna, Austria). A significance level of ≤0.05 was used for all subsequent analyses. The distribution of continuous variables was assessed visually with histogram and quantile plots. Normally distributed variables are expressed as mean ± standard deviation (SD), while skewed variables are expressed as median and interquartile range (IQR), and categorical variables are expressed as given number (*n*) and percentages. Baseline characteristics were described for all populations. Analyses of differences in baseline characteristics for two groups were assessed by unpaired *t*-test for normally distributed continuous variables, Mann–Whitney *U*-test for skewed continuous variables and *χ*^2^ test for categorical variables. Analyses of difference in baseline characteristics for three groups or more were assessed by ANOVA for normally distributed continuous variables, Kruskal–Wallis for skewed continuous variables, and *χ*^2^ test for categorical variables. Post hoc analyses for between-group differences were performed for ANOVA by Tukey's test, for Kruskal–Wallis by pairwise comparison with Dunn's test with Bonferroni correction for multiple testing and for *χ*^2^ by *z*-test for independent proportions with Bonferroni correction for multiple testing.

The current study was reported in line with the STrengthening the Reporting of OBservational studies in Epidemiology (STROBE) guidelines (see Supplementary material online, *Table S1*).^25^ The data underlying this article are available in the article and in its online Supplementary material. Missing data were reported separately for all variables of interest, and no further actions were performed regarding missing data.

### 2.5 Biomarker discovery analyses

In the biomarker discovery phase, serum samples of ATTRwt discovery patients were compared with serum samples of ATTRwt-negative discovery patients. Protein biomarkers were measured using multiplex proximity extension assays of the Olink target cardiovascular II, cardiovascular III, oncology II and immune response panels consisting of 92 proteins each (368 total). These panels were chosen to obtain a diverse as possible view on different aspects ATTRwt-CM, AL-CM, and HFpEF/HFmrEF in line with previous studies that successfully utilized these specific panels for biomarker identification in HF.^26,27^ The serum protein biomarkers measured are represented in relative concentrations of the serum protein expressed as Normalized Protein eXpression (NPX).^28^ Four protein biomarkers overlapped between panels, those being interleukin 6 (measured three times) and tissue factor pathway inhibitor 2, amphiregulin, and KIT ligand (all measured twice). For duplicate/triplicate measurements, the mean of all measurements was used instead. Thus, 363 unique protein biomarkers were available for further analyses.

The mixOmics package version 6.24.0 for R was used to perform sparse partial least squares discriminant analysis (sPLS-da),^29^ in order to visually represent the distribution ATTRwt discovery and ATTRwt-negative discovery patients, according to the circulating signatures of 363 unique protein biomarkers over the first two components. A model was fitted to the data with a 10-fold cross-validation averaged 50 times, and individuals were plotted on a sPLS-da plot with 95% confidence interval (CI) ellipses. Differential expression of the protein biomarkers was visually presented using a volcano plot, based on the results of univariable logistic regression for each protein biomarker, and the log_2_-fold change between groups. The threshold for statistical significance was adjusted for multiple testing based on the Benjamini–Hochberg false discovery rate method, and the family-wise error rate was controlled at 5% (false discovery rate ≤0.05). Pathway analyses were performed using g:Profiler and the Kyoto Encyclopaedia of Genes and Genomes.^30^ Significance was calculated with a Fisher's one-tailed test and corrected for multiple testing according to the Benjamini–Hochberg false discovery rate method.

The ncvreg package version 3.14.1 for R was used to perform a min-max concave penalty (MCP) penalized logistic regression with k-fold cross-validation, in order to select features that best discriminated between ATTRwt discovery and ATTRwt-negative discovery patients.^31^ All 363 unique protein biomarkers, as well as age, sex, a history of hypertension, diabetes mellitus type 2 and/or coronary artery disease (CAD) were included as possible features to account for baseline differences. The optimal value of the penalization term *λ* was determined as the value that minimized the cross-validation error in *k*-fold cross-validation. The reliability of selected features was evaluated using the built-in marginal false discovery rate (mFDR), and only features with mFDR ≤0.05 were considered significant in this analysis. The predictive capacity of the model was evaluated using cross-validated *R*^2^. We selected a Least Absolute Shrinkage and Selection Operator method for feature selection due to its robustness against noise and its ability to produce a sparser model with fewer non-zero coefficients compared with partial least squares (PLS), which aligns with our objective of identifying a small number of clinically relevant biomarkers.^32^ Specifically, we chose the MCP variant for its capacity to yield less biased estimates and sparser results.^33^

### 2.6 External biomarker validation analyses

External quantitative validation of the protein biomarkers selected from the protein biomarker discovery phase by MCP was measured with ELISA. ELISAs were performed to determine the concentration of decorin [DCN (ThermoFischer Scientific Inc, US, number EHDCNX5)], alpha-L-iduronidase [IDUA (ThermoFischer Scientific Inc, US, number EH247RBX5)] and human Galactosidase β-1 [GLB-1 (Novus: R&D systems, US, number NBP2–82181)] in selected serum samples. The assays were all performed according to the methods described by the manufacturers. The selected protein biomarkers were compared between ATTRwt validation patients and ATTRwt-negative validation patients. If not significantly different in concentrations between the groups, the biomarker was excluded from further analyses.

Prediction modelling was performed with logistic regression for the protein biomarker of interest to determine the association with ATTRwt, after the assumptions for logistic regression were controlled and met. The odds ratio (OR) and 95% CI were calculated. Receiver operation curves (ROC) were created with the logistic regression using pROC package version 1.18.5 for R.^34^ The AUC and 95% CI were calculated. A Youden point was calculated to determine the optimal cut-off point based on the highest combined sensitivity and specificity. The DeLong method was employed to calculate the differences between AUC.^35^ Linear regression analyses were performed to explore the correlations between known disease characteristics and the selected biomarkers.

## 3. Results

### 3.1 Biomarker discovery

**Figure 1:**
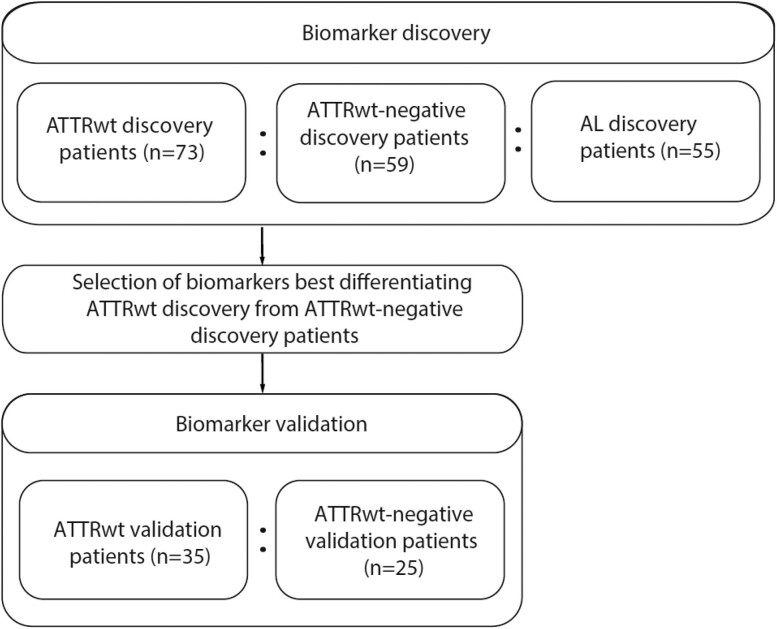
Flowchart of the study. ATTRwt, wild-type transthyretin amyloidosis with cardiomyopathy; AL, light chain; samples of patients included in prospective biomarker validation were not previously used in biomarker discovery.

A flowchart of the study design is presented in *Figure 1*. A full comparison of baseline characteristics of the discovery cohort is presented in *Table 1*. An overview of the missing data is presented in the Supplementary data (Supplementary material online, *Table S2*). A total of 73 ATTRwt discovery, 59 ATTRwt-negative, and 55 AL discovery patients were included for protein biomarker discovery analyses.

**Table 1:** Baseline characteristics of ATTRwt discovery patients (*n* = 73), ATTRwt-negative discovery patients (*n* = 59), and AL discovery patients (*n* = 55)

	ATTRwt discovery (*n* = 73)	ATTRwt-negative discovery (*n* = 59)	AL discovery (*n* = 55)	*P*-val.^a^	*P*-val.^b^	*P*-val.^c^	*P*-val.^d^
**Patient characteristics**							
Age, years	74 ± 7	72 ± 7	67 ± 9	***	n.s.	n.s.	***
Men, *n* (%)	67 (92)	31 (53)	36 (66)	***	*	*	n.s.
HT, *n* (%)	11 (15)	47 (80)	9 (16)	***	*	*	n.s.
DM II, *n* (%)	7 (10)	21 (36)	3 (6)	***	*	n.s.	*
CAD, *n* (%)	3 (4)	14 (24)	2 (4)	***	*	n.s.	*
**Functionality**							
NYHA class, *n* (%)				*	n.s.	n.s.	*
* I*	9 (12)	0	9 (16)				
* II*	42 (58)	38 (64)	24 (43)				
* III*	22 (30)	21 (36)	21 (38)				
* IV*	0	0	1 (2)				
**Medication**							
β-blocker, *n* (%)	47 (64)	49 (83)	25 (46)	***	*	n.s.	*
ACEi, *n* (%)	19 (26)	18 (31)	17 (31)	n.s.	n.s.	n.s.	n.s.
ARB, *n* (%)	6 (8)	13 (22)	1 (2)	***	*	n.s.	*
MRA, *n* (%)	38 (52)	21 (42)	14 (26)	*	n.s.	n.s.	*
Diuretics, *n* (%)	68 (93)	50 (86)	27 (49)	***	n.s.	*	*
**Laboratory values**							
hs-Troponin T	55 [40–75]	20 [14–35]	68 [22–115]	**	**	*	***
NT-proBNP (ng/L)	3127 [1826–5239]	1569 [740–2966]	2838 [602–9472]	*	***	n.s.	n.s.
eGFR (mL/min 1.73 m^2^)	59 ± 16	56 ± 23	61 ± 27	n.s.	n.s.	n.s.	n.s.
**Echocardiography**							
LVEF (%)	46 ± 10	54 ± 7	53 ± 11	***	***	***	n.s.
IVST (mm)	18 ± 3	11 ± 2	14 ± 4	***	***	***	***
LVMI (g/m^2^)	176 ± 52	100 ± 31	120 ± 44	***	***	***	n.s.

Significance is reported as ***≤0.001, **≤0.01, *≤0.05 and n.s. if >0.05. Data are presented as medians with IQRs, means with standard deviations, or number with percentages. Overall between-group differences are tested with ANOVA for normally distributed continuous variables, Kruskal–Wallis for non-normally distributed continuous variables and *χ*^2^ for categorical variables. Post hoc analyses were performed for ANOVA by Tukey's test, for Kruskal–Wallis by pairwise comparison with Dunn's test with Bonferroni correction and for *χ*^2^ by *z*-test for independent proportions with Bonferroni correction for multiple testing.

Val., value; *n*, number; HT, hypertension; DMII, diabetes mellitus type 2; CAD, coronary artery disease in medical history; ACEi, angiotensin-converting enzyme inhibitor; ARB, angiotensin receptor blocker; MRA, mineral corticoid receptor antagonist; Diuretics consist of loop- and thiazide diuretics; hs, high sensitivity; NT-proBNP, N-terminal prohormone brain natriuretic peptide; eGFR, estimated glomerular filtration rate; LVEF, left ventricular ejection fraction; IVST, interventricular septal thickness at diastole; LVMI, left ventricular mass index.

^a^Between-group differences.

^b^Between ATTRwt discovery and ATTRwt-negative discovery.

^c^Between ATTRwt discovery and AL discovery.

^d^Between AL discovery and ATTRwt-negative discovery.

#### 3.1.1 ATTRwt discovery patients compared with ATTRwt-negative discovery patients

ATTRwt discovery patients were older, predominantly male, had fewer comorbidities and higher hs-Troponin T and NT-proBNP levels. On echocardiography LVEF was decreased, and intraventricular septal thickness (IVST) and left ventricular mass index (LVMI) were increased in ATTRwt discovery patients.

**Figure 2:**
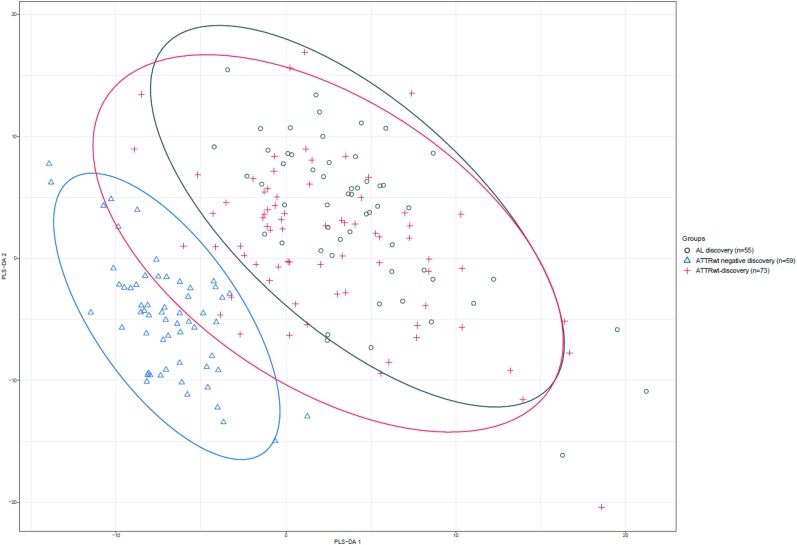
sPLS-da sample plot visualizing the first two components with a selection of the 363 unique protein biomarkers, differentiating ATTRwt discovery patients (*n* = 73), ATTRwt-negative discovery patients (*n* = 59), and AL discovery patients. The 95% CI ellipses are the corresponding colours. Included biomarkers in the first two components can be viewed in Supplementary material online, *Figure S1*.

**Figure 3:**
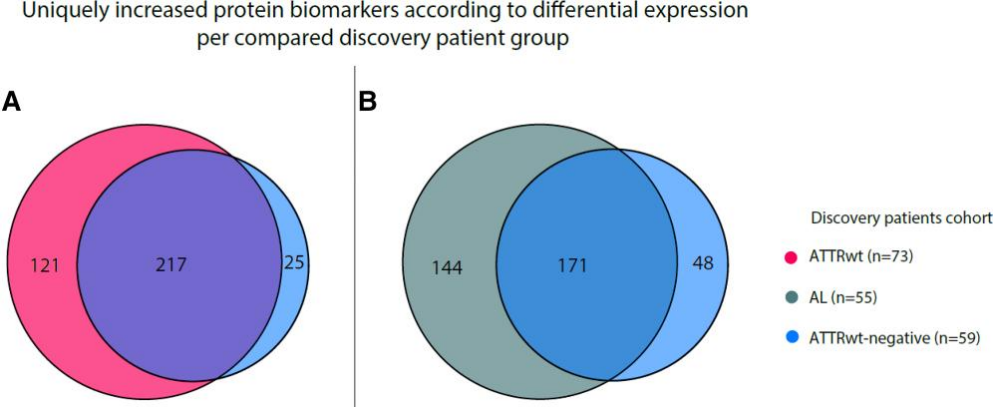
Uniquely increased serum protein biomarkers for the different combinations in the discovery analyses, based on the *P*-value adjusted for multiple testing by Benjamini-Hochberg false discovery rate method. A total of 363 unique protein biomarkers were measured by multiplex proximity extension assays. No biomarkers were significantly increased or decreased between ATTRwt and AL discovery patients, and therefore, no figure is shown.

An sPLS-da analysis showed considerable visual differentiation between ATTRwt discovery and ATTRwt-negative discovery patients (*Figure 2*). Differential expression of the individual protein biomarkers was visualized and a total of 121 protein biomarkers were uniquely increased in ATTRwt discovery patients (*Figure 3*, Supplementary material online, *Figure S2*; Supplementary material online, *Table S4*). The pathways of cell adhesion molecules and proteoglycans (PG) in tumour microenvironments were significantly associated with ATTRwt (adjusted *P*-value of 0.007 and 0.045, respectively; see Supplementary material online, *Figure S3*). A total of 25 protein biomarkers were uniquely decreased in ATTRwt discovery patients, but no significant down-regulated pathways were identified.

MCP identified three protein biomarkers that discriminated optimally between ATTRwt discovery and ATTRwt-negative discovery patients, independent of age, sex, and a history of hypertension, diabetes mellitus or CAD (*R*^2^ 0.71, signal to noise ratio 2.43, prediction error 0.000; see Supplementary material online, *Figure S4* and *Table S6*). The three selected, significantly differentiating, biomarkers were the serum protein biomarkers DCN, IDUA and GLB-1 [adjusted *P*-value 3.5 × 10^−4^, OR 38.6; adjusted *P*-value 0.0017, OR 0.13; adjusted *P*-value 0.029, OR 3.92, respectively (see Supplementary material online, *Table S7*)]. A visual representation of the NPX-values of the selected biomarkers compared between the groups is presented in Supplementary material online, *Figure S5*.

#### 3.1.2 ATTRwt discovery patients compared with AL discovery patients

AL discovery patients were more often male and of non-significantly lower age; they had higher hs-Troponin T levels than ATTRwt-CM discovery patients and comparable NT-proBNP levels. Additionally, they exhibited lower values for echocardiographic parameters, such as LVEF, IVST, and LVMI. sPLS-da showed visual overlap between both cohorts, no significantly different biomarkers were identified by differential expression analyses, and no biomarkers were selected by MCP that could differentiate ATTRwt-CM from AL-CM based on the 363 unique protein biomarkers.

#### 3.1.3 AL discovery patients compared with ATTRwt-negative discovery patients

AL discovery patients were younger with a low prevalence of comorbidities and worse New York Health Association (NYHA) functional class compared with ATTRwt-negative patients, with an increased IVST and LVMI. sPLS-da, differential expression analysis and pathway analyses showed similar results as ATTRwt compared with ATTRwt-negative discovery patients (*Figure 3*, Supplementary material online, *Figures S1–S3* and *Table S4*). MCP identified three protein biomarkers that discriminated optimally between AL discovery and ATTRwt-negative discovery patients, being ADAM Metallopeptidase With Thrombospondin Type 1 Motif 15 (ADAMTS 15), Fibroblast Growth Factor Binding Protein 1 (FGB-BP1) and DCN [*R*^2^ 0.71, signal to noise ratio 2.44, prediction error 0.022 (Adjusted *P*-value 5.3 × 10^−5^, OR 0.13; adjusted *P*-value 4.1 × 10^−4^, OR 4.36 and adjusted *P*-value 6.0 × 10^−4^, OR 4.95] (see Supplementary material online, *Table S7*).

### 3.2 External biomarker validation

A full comparison of baseline characteristics of the prospective validation cohort is presented in *Table 2*. A total of 35 ATTRwt validation patients and 25 ATTRwt-negative validation patients, with similar age and a male predominance, were included for external biomarker validation. Both groups had comparable NT-proBNP levels but ATTRwt validation patients exhibited higher Troponin-T and estimated glomerular filtration rate (eGFR). On echocardiography, LVEF was similar in both groups but IVST and LVMI were increased in ATTRwt validation patients. Additional information specific to the ATTRwt discovery patients is presented in Supplementary material online, *Table S8*. Known comorbidities such as carpal tunnel syndrome were frequent, most patients were NYHA-functional class II and National Amyloidosis Center stage II, and global longitudinal systolic strain was decreased compared with normal and most patients had Perugini grade ≥2 cardiac tracer uptake. ATTRwt patients in both cohorts were comparable apart from a higher hs-Troponin T and lower LVEF in discovery patients, as well for ATTRwt-negative patients in both cohorts apart from a lower eGFR in the prospective validation cohort (see Supplementary material online, *Tables S9* and *S10*).

**Table 2:** Baseline characteristics of ATTRwt validation patients vs. ATTRwt-negative validation patients

	ATTRwt validation (*n* = 35)	ATTRwt-negative validation (*n* = 25)	*P*-value
**Patient characteristics**			
Age, years	77 ± 6	74 ± 10	0.13
Men, *n* (%)	30 (86)	15 (60)	**<0.001**
HT, *n* (%)	15 (43)	21 (84)	**0.002**
DM II, *n* (%)	6 (17)	12 (48)	**0.008**
CAD, *n* (%)	3 (9)	4 (16)	0.36
**Functionality**			
NYHA class, *n* (%)			0.21
* I*	2 (6)	0	
* II*	24 (69)	14 (56)	
* III*	8 (23)	11 (44)	
* IV*	1 (3)	0	
**Medication**			
β-blocker, *n* (%)	21 (60)	18 (82)	0.16
ACEi, *n* (%)	14 (40)	5 (23)	0.14
ARB, *n* (%)	9 (26)	4 (18)	0.55
MRA, *n* (%)	15 (43)	10 (46)	0.94
Diuretics, *n* (%)	19 (54)	20 (91)	**0.003**
**Laboratory values**			
hs-Troponin T, ng/L	46 [32–57]	28 [24–49]	0.07
NT-proBNP, ng/L	2044 [1201–3434]	1665 [948–3848]	0.26
eGFR, mL/min 1.73 m^2^	62 ± 18	45 ± 17	**<0.001**
**Echocardiography**			
LVEF, %	51 ± 10	52 ± 5	0.78
IVST, mm	17 ± 3	12 ± 3	**<0.001**
LVMI, g/m^2^	166 ± 59	101 ± 30	**<0.001**

Data are presented as mean ± SD, median (IQR), or number (%). Significance was calculated following an unpaired *t*-test for normally distributed continuous variables and Mann–Whitney *U*-test for skewed continuous variables. Significant values are highlighted in bold.

*n*, number; HT, hypertension; DMII, diabetes mellitus type 2; CAD, coronary artery disease; ACEi, angiotensin-converting enzyme inhibitor; ARB, angiotensin receptor blocker; MRA, mineral corticoid receptor antagonist; hs, high sensitivity; eGFR, estimated glomerular filtration rate; LVEF, left ventricular ejection fraction; IVST, intraventricular septal thickness; LVMI, left ventricular mass index.

The results of the ELISAs are presented in *Table 3* and Supplementary material online, *Figure S6*. The mean levels of DCN were, in line with the discovery cohort, significantly increased in ATTRwt validation patients, compared with ATTRwt-negative validation patients (4063 ± 1246 pg/mL vs. 3103 ± 827 pg/mL; *P* = 0.001). The median levels of IDUA were, in line with the discovery cohort, significantly decreased in ATTRwt validation patients, compared with ATTRwt-negative validation patients [2.04 ng/mL (1.33–3.00) vs. 5.12 ng/mL (2.70–5.78); *P* < 0.001]. The mean levels of GLB-1, as opposed to an increase in ATTRwt discovery patients, did not differ between ATTRwt validation patients and ATTRwt-negative validation patients (55 514 ± 23 819 pg/mL vs. 69 524 ± 33 549 pg/mL; *P* = 0.06).

**Table 3:** Biomarker validation results in ATTRwt validation vs. ATTRwt-negative validation patients

Biomarker	ATTRwt validation (*n* = 35)	ATTRwt-negative validation (*n* = 25)	*P*-value
DCN, pg/mL	4063 ± 1246	3103 ± 827	**0.001**
IDUA, ng/mL	2.04 [1.33–3.00]	5.12 [2.70–5.78]	**<0.001**
GLB-1, pg/mL	55 514 ± 23 819	69 524 ± 33 549	0.06

Data are presented as mean ± SD, median (IQR), or number (%). Significance was calculated following an unpaired *t*-test for normally distributed continuous variables and Mann–Whitney *U* test for skewed continuous variables. Significant values are highlighted in bold.

**Figure 4:**
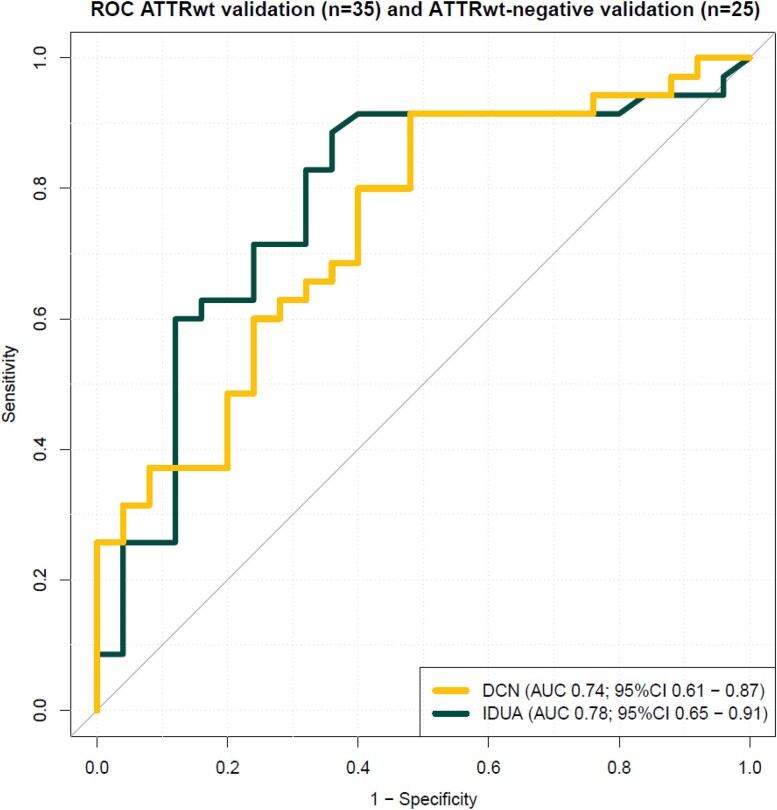
ROC curve for DCN and IDUA for predicting ATTRwt (*n* = 35) from ATTRwt-negative patients (*n* = 25) in the biomarker prospective validation cohort. ROC, receiver operating characteristics; AUC, area under the curve; CI, confidence interval.

DCN and IDUA were significant predictors of ATTRwt in logistic regression [OR, 3.3; 95% CI, 1.6–8.0 and OR, 0.4; 95% CI, 0.2–0.7, respectively (*Table 4*)]. After correction for IVST and LVMI, DCN remained a significant predictor, IDUA not. ROC curves for DCN and IDUA are presented in *Figure 4*. The AUC for DCN was 0.74 (95% CI, 0.61–0.87) with a sensitivity of 0.91 and specificity of 0.52 at a Youden point of ≥2895 pg/mL (see Supplementary material online, *Table S11*). The AUC for IDUA was 0.78 (95% CI, 0.65–0.91) with a sensitivity of 0.91 and specificity of 0.64 at a Youden point of <3.61 ng/mL. Both resulted in a lower number needed to scan (see Supplementary material online, *Table S11*).

**Table 4:** Addition of conventional disease markers on echocardiography in logistic regression to protein biomarker measurements as measured in ATTRwt validation (*n* = 35) and ATTRwt-negative validation (*n* = 25) patients

Biomarker	OR	95% CI	*P*-value
DCN	3.3	(1.6–8.3)	**0.004**
DCN + IVST	2.9	(1.2–8.9)	**0.03**
DCN + LVMI	3.1	(1.4–9.7)	**0.02**
IDUA	0.4	(0.2–0.7)	**0.01**
IDUA + IVST	0.7	(0.3–1.5)	0.45
IDUA + LVMI	0.8	(0.3–1.8)	0.60

Test are performed according logistic regression with correction for added variable, reported odds ratios are for the respective biomarker (DCN or IDUA) when corrected for the additional echocardiography parameters. ATTRwt validation patients (*n* = 35) are compared with ATTRwt-negative validation patients (*n* = 25). Significant values are highlighted in bold.

OR, odds ratio; CI, confidence interval; IVST, intraventricular septal thickness; LVMI, left ventricular mass index.

DCN and IDUA were significantly better at predicting ATTRwt than NT-proBNP (AUC, 0.57; 95% CI, 0.41–0.72; *P* = 0.05 and *P* = 0.02, respectively). DCN was not significantly better at predicting ATTRwt than hs-Troponin T (AUC, 0.61; 95% CI, 0.45–0.75, *P* = 0.16), whereas IDUA was (*P* = 0.04). A combination of DCN and IDUA was not significantly better than the individual biomarkers [AUC 0.82; 95% CI 0.71–0.93 (see Supplementary material online, *Figure S7*)]. A single and multivariable logistic model of the three markers most significant per category in univariable logistic regression (sex, eGFR, and IVST) was created and presented in *Table 5*, the multivariable model had an AUC of 0.92 (95% CI 0.85–0.98) with a sensitivity of 0.94 and specificity of 0.76 (see Supplementary material online, *Figure S7*). This model differentiated significantly better between ATTRwt validation and ATTRwt-negative validation patients than DCN (*P* = 0.008), but this was not significantly different for IDUA (*P* = 0.07) or the combined DCN and IDUA model (*P* = 0.11).

**Table 5:** Univariable analyses for determination of optimal clinical multivariable model to differentiate ATTRwt validation (*n* = 35) and ATTRwt-negative validation (*n* = 25) patients

Univariable logistic regression			
Variable	OR	95% CI	*P*-value
Female sex	0.11	(0.03–0.36)	**<0.001**
Hypertension	0.14	(0.04–0.47)	**0.003**
Diabetes mellitus	0.22	(0.07–0.70)	**0.02**
Diuretics	0.38	(0.11–1.18)	0.10
eGFR	1.06	(1.02–1.09)	**0.002**
IVST	1.69	(1.34–2.25)	**<0.001**
LVMI	1.04	(1.03–1.08)	**<0.001**

Multivariable logistic regression			
Variable	OR	95% CI	*P*-value
Female sex	0.25	(0.04–1.27)	0.10
eGFR	1.05	(1.00–1.11)	**0.03**
IVST	1.62	(1.26–2.27)	**<0.001**

Test are performed according logistic regression with correction for added variable, reported odds ratios are for the respective biomarker (DCN or IDUA) when corrected for the additional echocardiography parameters. ATTRwt validation patients (*n* = 35) are compared with ATTRwt-negative validation patients (*n* = 25). A maximum number of three variables was selected for the clinical model, based on the minimum number of observations of 10 per added variable. The three variables for multivariable logistic regression were selected on the lowest *P*-values per category. Significant values are highlighted in bold.

OR, odds ratio; CI, confidence interval.

DCN nor IDUA were significantly associated with age, hs-Troponin T, NT-proBNP, eGFR, LVEF, IVST or LVMI within ATTRwt or ATTRwt-negative validation patients (see Supplementary material online, *Table S12*).

## 4. Discussion

In our study we demonstrated that ATTRwt has a distinct biomarker profile compared with ATTRwt-negative patients, confirming the difference between ATTRwt amyloidosis and other types of HFpEF and HFmrEF.^36^ We established that the distinction is most likely a general characteristic of cardiac amyloidosis, based on the overlapping profiles and commonly increased pathways in ATTRwt-CM and AL-CM, rather than an ATTRwt-CM-specific mechanism. We identified two significantly up-regulated related pathways in cardiac amyloidosis (ATTRwt-CM and AL-CM) compared with ATTRwt-negative HFpEF/HFmrEF and showed individual biomarkers related to extracellular matrix (ECM) handling to be elevated in cardiac amyloidosis.

DCN, GLB-1, and IDUA were validated out of 363 unique protein biomarkers with the potential to differentiate ATTRwt patients from ATTRwt-negative patients. DCN and IDUA were significantly different in ATTRwt-CM in prospective validation and provided moderate predictive capabilities. Based on the high sensitivity of IDUA and DCN and the absence of correlation with existing disease markers, these biomarkers could be of potential benefit as an initial screening tool for excluding patients from having ATTRwt-CM in patients with HFpEF/HFmrEF, lowering the number needed to scan. The identified patients can subsequently be diagnosed accurately according to the non-invasive standard. It is important to note that DCN was inferior to a clinical model based on sex, eGFR, and IVST in the prediction of ATTRwt-CM within our population, and after correction for IVST and LVMI, IDUA was no longer predictive of ATTRwt-CM. However, as in early ATTRwt-CM, these echocardiographic markers deteriorate less, and without correlation with existing disease markers, DCN and IDUA could prove beneficial early detection of ATTRwt-CM.

Interestingly, from a pathophysiological point of view, with regard to the selected biomarkers for differentiation of ATTRwt-CM and AL-CM from ATTRwt-negative HFpEF/HFmrEF, DCN, IDUA, GLB-1, and ADAMTS-15 are all constituents of the ECM. DCN is a small leucine proteoglycan (PG) in the ECM and present in a wide variety of organs and involved in multiple processes in the heart. The role of DCN in amyloidosis pathogenesis remains unclear. On one hand, on a cardiac level, it might provide a protective function through inhibition of cardiac dysfunction via the Tissue Growth Factor Beta pathway suppression and reducing hypertrophy through reduced ECM remodelling.^37^ On the other hand, studies suggest a systemic pro-amyloidogenic role for PG, including DCN, and glycosaminoglycans (GAGs), including co-localization with amyloid deposits and potential for promoting fibrillization and accelerating amyloid aggregation.^38^ PGs co-localize with amyloid deposits and PGs and GAGs are found to be deposited at amyloid binding spots prior to amyloid deposition.^39^ DCN has been identified as a constituent of the amyloid-specific proteome of ATTRwt-CM previously.^40^

Less is known about IDUA and no previous associations with amyloidosis have been reported. Compellingly, however, IDUA is a lysosomal hydrolase responsible for the degradation of GAGs dermatan sulfate and heparan sulfate.^41^ An increased heparan sulfate has been found in amyloidosis.^39^ The increased presence of the PG DCN, combined with the decreased lysosomal hydrolase IDUA, indicates an increase in PGs and GAGs in ATTRwt-CM and AL-CM and a decrease in clearing, as supported by the up-regulated PG pathway in ATTRwt-CM and AL-CM found in this study.

A decreased ADAMTS 15 in AL-CM and ATTRwt-CM compared with ATTRwt-negative patients is notable in combination with the increased expression of matrix metalloproteinases (MMPs). MMPs play a role in collagen degradation which is part of the ECM homeostasis. MMP9 was previously found to be increased in AL-CM as well as in ATTRwt-CM.^42^ Our study aligns with the increase of MMP9 and additionally MMP2 in AL-CM and ATTRwt-CM.

While we identified enriched pathways related to cell adhesion and the tumour microenvironment, their relevance to ATTRwt-CM and AL-CM remains to be elucidated. The significant up-regulation of these pathways in both ATTRwt-CM and AL-CM compared with ATTRwt-negative HFpEF/HFmrEF patients suggests a common underlying mechanism for cardiac amyloidosis based on the selected serum protein biomarkers, instead of being a process specific to ATTRwt-CM. The association is most likely based on a systemic process in other tissues, rather than specific to the myocardium and the remodelling process, further supported by the lack of correlation between the identified biomarkers and established cardiac disease markers.

Other biomarkers identified in the biomarker discovery phase have been described in literature with regard to ATTRwt-CM, AL-CM, or HFpEF, but were not of attributed value to the MCP. Most notable is the significantly increased Hepatocyte Growth Factor (HGF) in ATTRwt and AL discovery patients. HGF was previously described as elevated in both ATTR and AL amyloidosis,^19^ but lacked diagnostic value in ATTRwt discovery patients and was not selected by our MCP analyses.

This study has several limitations. No conclusions regarding causality of the identified protein biomarkers can be made based on the current study and no inferences can be gained in the myocardium based on the serum proteins. This is a single-centre study and the generalizability to other cohorts may be limited. The overall sample size of this study is small compared with similar studies in other fields. Baseline differences between discovery and validation, ATTRwt and ATTRwt-negative patients were prevalent, a characteristic unfortunately inherently associated with the nature of ATTRwt-CM in currently diagnosed stages compared with ATTRwt-negative patients. The performed multiplex proximity extension assays commercially available at the time of the conduction of the discovery phase only cover a small portion of the proteome. The inclusion of additional proteins may have allowed the identification of more meaningful pathway enrichment. No advanced HFpEF workup such as echo stress tests or invasive haemodynamic measurements was performed in our study. In the current study, a relative prevalence of ATTRwt-CM of 58% does not reflect a realistic prevalence in a cohort, therefore no positive predictive and negative predictive values were reported in the main text. Echocardiographic wall thickness and mass parameters for the discovery cohort should be interpreted with caution because of the percentage of missing data. Lastly, no follow-up blood sampling was available to determine the use of biomarkers in the identification of potential responders vs. non-responders to disease-modifying therapies.

To determine the clinical relevance of DCN and IDUA in the screening for ATTRwt-CM in patients with HFpEF/HFmrEF, a larger, prospective study would need to be conducted. Expanding research on protein biomarkers to assess the potential to distinguish patients with ATTRwt-CM from patients with severe aortic stenosis^43^ or pre-selected patients with carpal tunnel syndrome, could provide valuable insights.

The applicability of DCN and IDUA in pre-symptomatic or early disease stage ATTRwt-CM could be further explored, given the absence of correlation to existing ATTRwt-CM disease parameters. The value of DCN and IDUA in monitoring disease progression or therapy response is of interest. It is crucial to account for potential interference from other diseases as well in the context of clinical implementation.^44^ Further research is needed to deepen the pathophysiological understanding of ATTRwt-CM to elucidate the significance of the increased pathways and other biomarkers. PG and GAGs and other ECM proteins, beyond the current panels in our study, seem compelling to investigate.

**Translational Perspective**

There is a clinical unmet need for biomarkers for the screening of ATTRwt-CM in at-risk populations. We identified biomarkers DCN and IDUA for this purpose. This approach could streamline ATTRwt-CM diagnosis by adding to current screening methods, potentially leading to earlier intervention and improved patient outcomes. Further validation and integration of these markers are necessary.

Secondly, our study reveals a unique biomarker signature in wild-type transthyretin cardiomyopathy (ATTRwt-CM) and light chain (AL)-CM compared with ATTRwt-negative HF with preserved and mildly reduced ejection fraction (HFpEF/HfmrEF). The identified differentiating biomarkers rather reflect a systemic process related to cardiac amyloidosis in general, than disease-specific (i.e. ATTRwt-CM and AL-CM).

## 5. Conclusion

ATTRwt-CM has a distinctly different biomarker profile compared with HFpEF/HFmrEF, while ATTRwt-CM patients share a similar biomarker profile with AL-CM patients characterized by up-regulation of PG and cell-adhesion pathways. The biomarkers DCN and IDUA show the potential to serve as an initial screening tool.

The identified biomarkers may represent a distinct disease mechanism or response, independent of conventional disease markers. Further research is needed to determine the clinical usefulness of these and other ECM components in identifying ATTRwt-CM, as well as for potential treatment targets or monitoring purposes.

## Supplementary Material

cvae189_Supplementary_Data
