# Supplementary material for: Circulating ECM proteins decorin and alpha-L-iduronidase differentiate ATTRwt-CM from ATTRwt-negative HFpEF/HFmrEF
Source: Cardiovasc Res. 2024 Sep 17;120(14):1727–36. doi: 10.1093/cvr/cvae189 (PMC11587557; doi:10.1093/cvr/cvae189)
Supplement: cvae189_Supplementary_Data [file cvae189_supplementary_data.docx]

# Supplementary figures

| **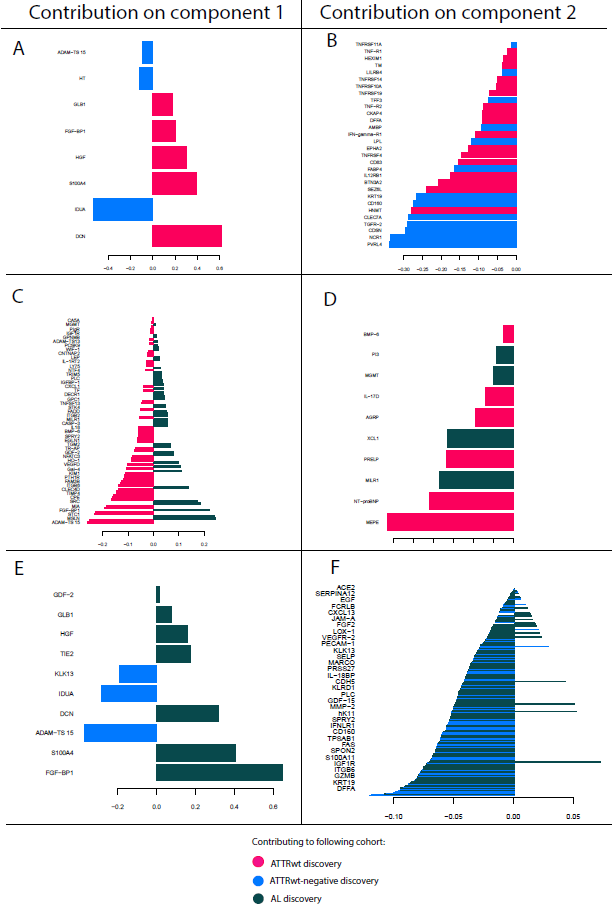** |
| --- |
| *Figure S1.* Contribution of biomarkers to the components 1 and 2 of the sPLS-da, selected from 363 unique protein biomarkers as measured by multiplex proximity extension assays in ATTRwt discovery patients (n=73) ATTRwt-negative discovery patients (n=59) and AL discovery patients (n=55). Discovery patients from the ATTRwt cohort were compared to ATTRwt-negative cohort (A,B); ATTRwt compared to AL (C,D); AL compared to ATTRwt-negative (E,F) and ATTRwt combined with AL with ATTRwt-negative (G,H). |

| 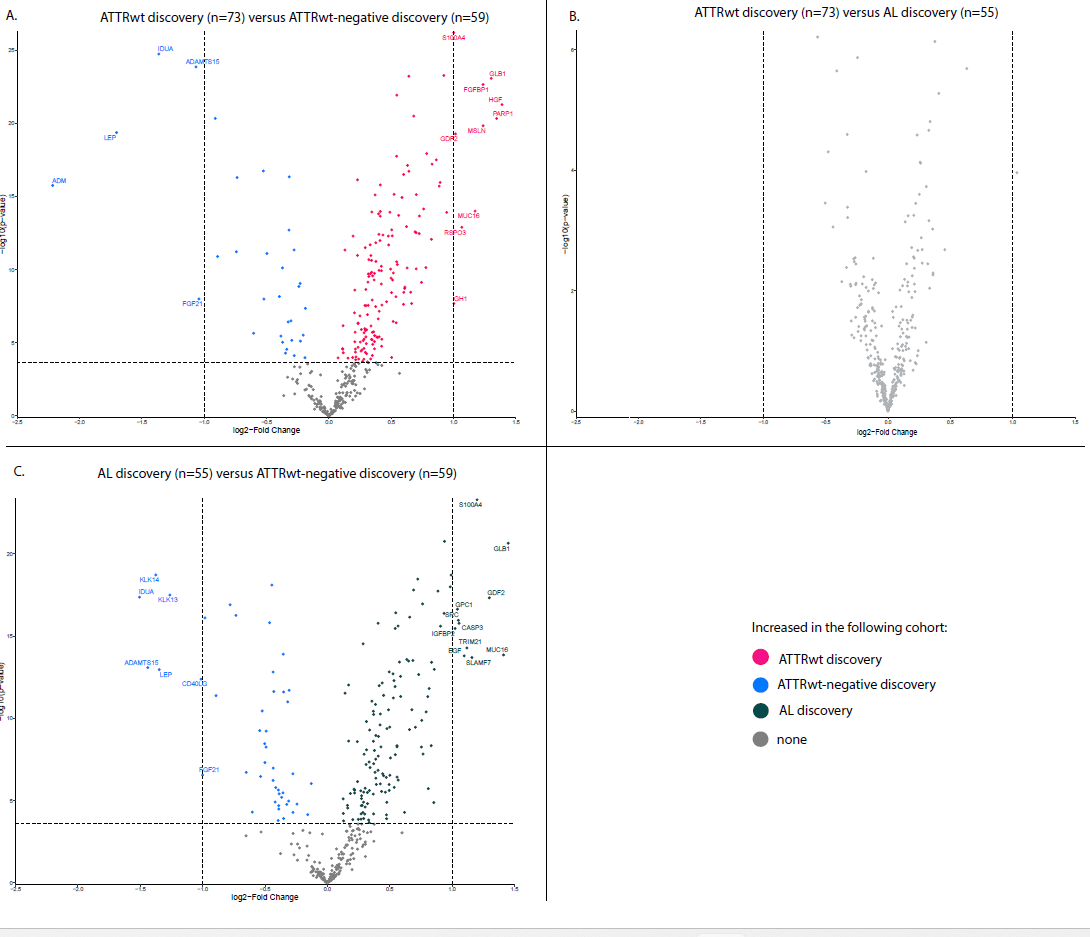 |
| --- |
| *Figure S2.* Differential expression analysis of 363 unique protein biomarkers comparing different cohorts for increased expression of protein biomarkers. Significance is reported according to the univariable logistic regression, the threshold for statistical significance was adjusted for multiple testing based on the Benjamini-Hochberg false discovery rate method. |

| 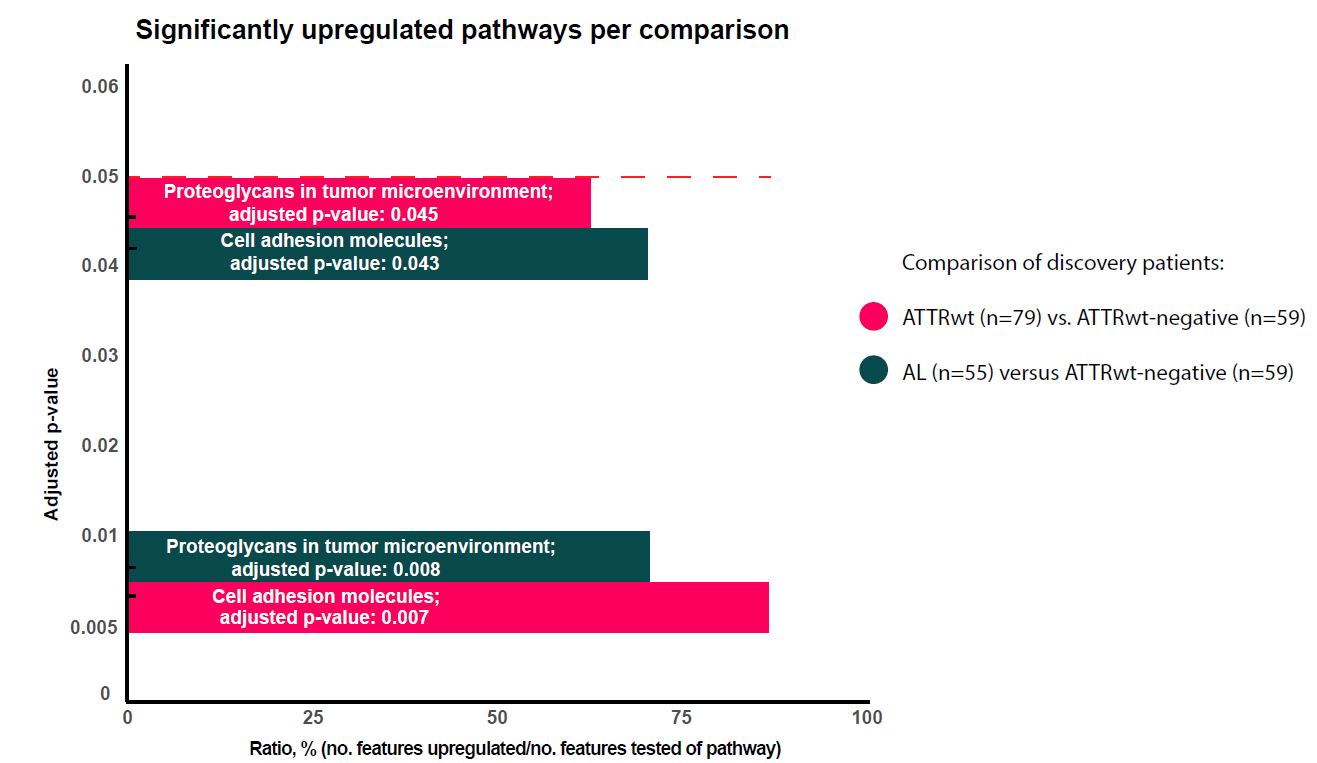 |
| --- |
| *Figure S3.* Significantly upregulated KEGG pathways per cohort comparison based on differential expression analysis results of 363 unique protein biomarkers as measured per multiplex proximity extension assays. KEGG pathway analyses were performed in g:Profiler with a correction for multiple testing according to Benjamini-Hochberg false discovery rate method. On the x-axis the ratio of upregulated features compared to the total number of features tested is represented as a percentage. |

| **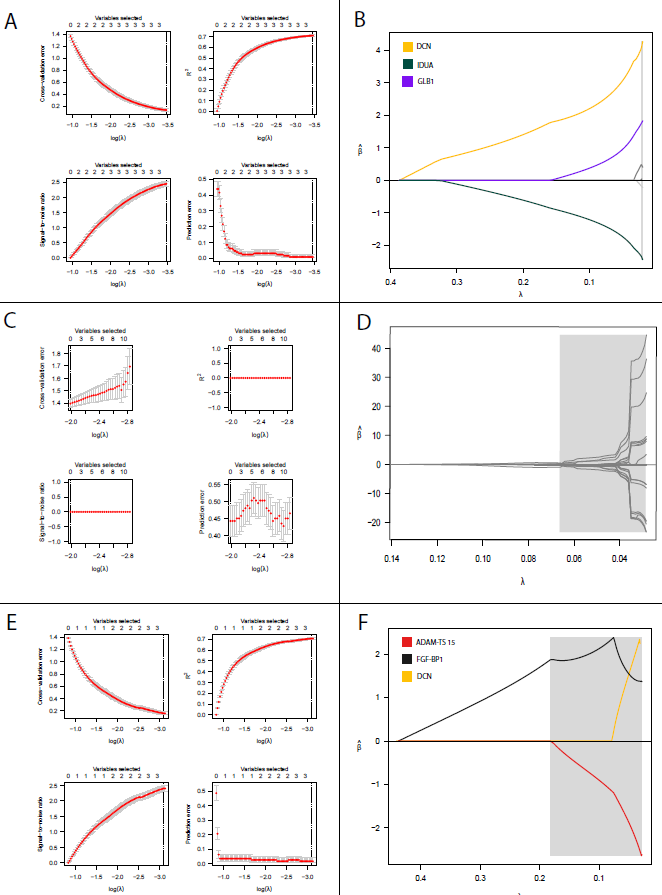** |
| --- |
| *Figure S4.* Determination of optimal number of biomarkers using penalised regression with min-max concave penalty (MCP) penalised regression based on multiplex proximity extension assays of 363 unique protein biomarkers. Selection in ATTRwt discovery patients (n=73) compared to ATTRwt-negative discovery patients (n=59) (A,B). Selection in ATTRwt discovery patients (n=73) compared to AL discovery patients (n=55) (C,D). Selection in AL discovery patients (n=55) compared to ATTRwt-negative discovery patients (n=59) (E,F). Selection in ATTRwt and AL discovery patients (n=73, n=55) compared to ATTRwt-negative discovery patients (n=59) (G,H). |

| *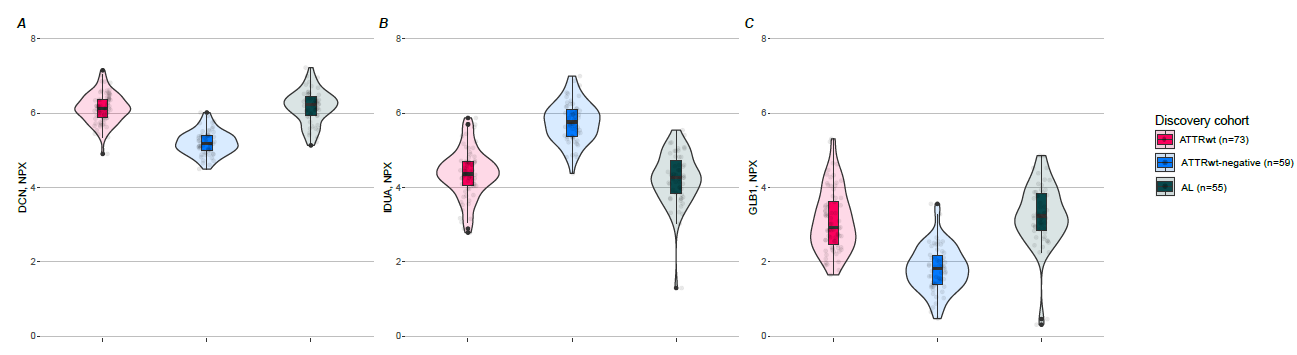* |
| --- |
| *Figure S5.* Violin plots showing Normalised Protein eXpression values as measured by multiplex proximity extension assays for the selected biomarkers comparing ATTRwt discovery patients (n=73), ATTRwt-negative discovery patients (n=59) and AL discovery patients (n=55). A violin plot in the background shows the distribution density visually, a dot plot shows the distribution of the measured values individually and a boxplot shows the median and interquartile range for DCN (A), IDUA (B) and GLB-1 (C). |

| *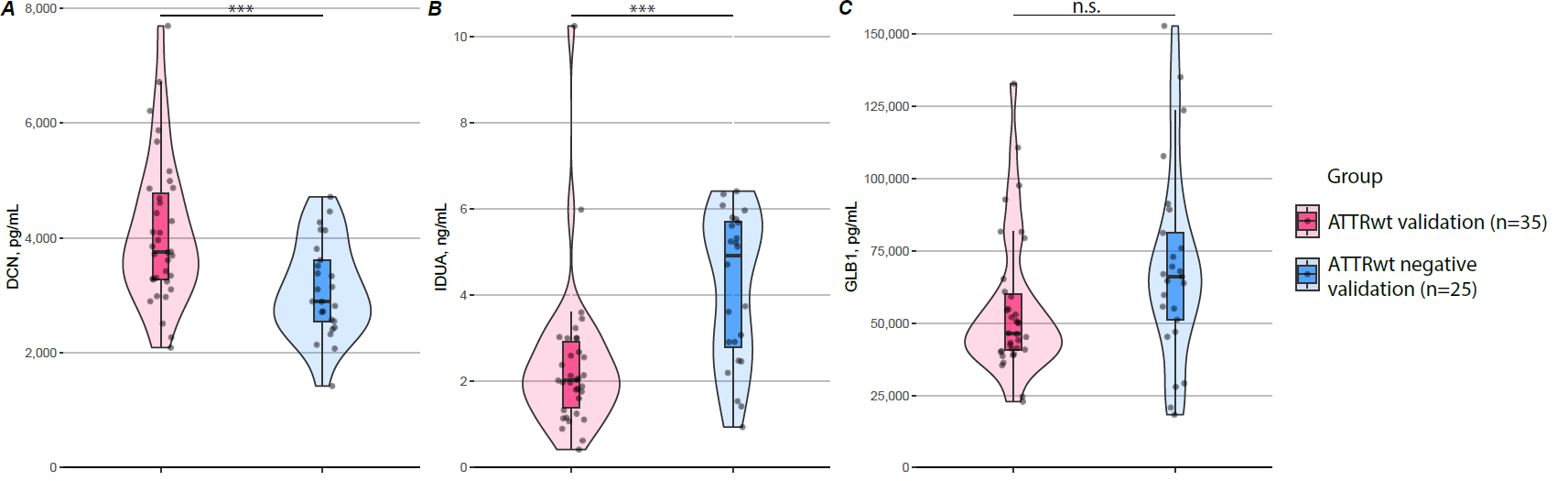* |
| --- |
| *Figure S6.* Violin plots showing distribution of biomarkers of ATTRwt validation patients (n=35) and ATTRwt-negative validation patients (n=25). The violin plot in the background shows the distribution density visually, a dot plot shows the distribution of the measured values individually and a boxplot shows the median and interquartile range for DCN (A), IDUA (B) and GLB-1 (C). Significance was calculated following an unpaired t-test for normally distributed continuous variables and Mann-Whitney U test for skewed continuous variables; ***, p ≤0.001; **, p ≤0.01, *≤0.05, n.s., not significant. |

| 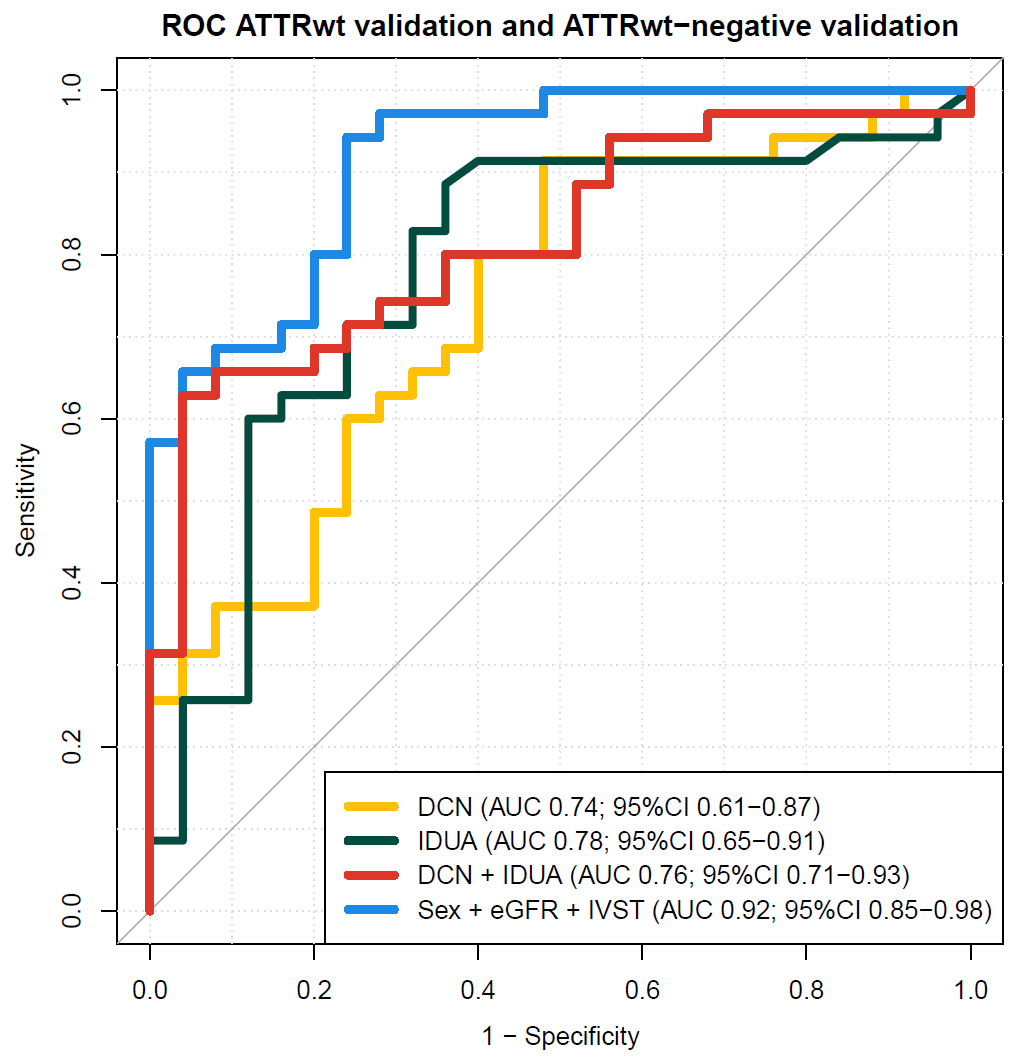 |
| --- |
| *Figure S7.* ROC curve for DCN, IDUA, combined and a clinical model for predicting ATTRwt (n=35) from ATTRwt-negative patients (n=25) in the biomarker prospective validation cohort. ROC, receiver operating characteristics; AUC, area under the curve; CI, confidence interval. |

Supplementary Tables

*Table S1.* STROBE Statement - Checklist of items that should be included in reports of *case-control studies*

|  | Item No | Recommendation | Page No |
| --- | --- | --- | --- |
| **Title and abstract** | 1 | (*a*) Indicate the study’s design with a commonly used term in the title or the abstract | 2 +  Graphic abstract |
|  |  | (*b*) Provide in the abstract an informative and balanced summary of what was done and what was found | 2, 3 |
| Introduction | | | |
| Background/  rationale | 2 | Explain the scientific background and rationale for the investigation being reported | 2, 5, 6 |
| Objectives | 3 | State specific objectives, including any prespecified hypotheses | 5, 6 |
| Methods | | | |
| Study design | 4 | Present key elements of study design early in the paper | 2, 7-12 |
| Setting | 5 | Describe the setting, locations, and relevant dates, including periods of recruitment, exposure, follow-up, and data collection | 7 |
| Participants | 6 | (*a*) Give the eligibility criteria, and the sources and methods of case ascertainment and control selection. Give the rationale for the choice of cases and controls | 7, 8 |
|  |  | (*b*) For matched studies, give matching criteria and the number of controls per case | - |
| Variables | 7 | Clearly define all outcomes, exposures, predictors, potential confounders, and effect modifiers. Give diagnostic criteria, if applicable | 8-12 |
| Data sources/ measurement | 8* | For each variable of interest, give sources of data and details of methods of assessment (measurement). Describe comparability of assessment methods if there is more than one group | 7-12 |
| Bias | 9 | Describe any efforts to address potential sources of bias | 9-12 |
| Study size | 10 | Explain how the study size was arrived at | 7, 8 |
| Quantitative variables | 11 | Explain how quantitative variables were handled in the analyses.  If applicable, describe which groupings were chosen and why | 8-12  - |
| Statistical methods | 12 | (*a*) Describe all statistical methods, including those used to control for confounding | 8-12 |
|  |  | (*b*) Describe any methods used to examine subgroups and interactions | - |
|  |  | (*c*) Explain how missing data were addressed | 9 |
|  |  | (*d*) If applicable, explain how matching of cases and controls was addressed | - |
|  |  | (*e*) Describe any sensitivity analyses | - |

| Results | | | |
| --- | --- | --- | --- |
| Participants | 13* | (a) Report numbers of individuals at each stage of study—eg numbers potentially eligible, examined for eligibility, confirmed eligible, included in the study, completing follow-up, and analysed | 35 |
|  |  | (b) Give reasons for non-participation at each stage | - |
|  |  | (c) Consider use of a flow diagram | 35 |
| Descriptive data | 14* | (a) Give characteristics of study participants (eg demographic, clinical, social) and information on exposures and potential confounders | 13-15 39-41 ,  S33,35 |
|  |  | (b) Indicate number of participants with missing data for each variable of interest | S14, S32, |
| Outcome data | 15* | Report numbers in each exposure category, or summary measures of exposure | 13-15 |
| Main results | 16 | (*a*) Give unadjusted estimates and, if applicable, confounder-adjusted estimates and their precision (eg, 95% confidence interval). Make clear which confounders were adjusted for and why they were included | 11, 13-15 |
|  |  | (*b*) Report category boundaries when continuous variables were categorized | 16 |
|  |  | (*c*) If relevant, consider translating estimates of relative risk into absolute risk for a meaningful time period | - |
| Other analyses | 17 | Report other analyses done—eg analyses of subgroups and interactions, and sensitivity analyses | 13-16 |
| Discussion | | | |
| Key results | 18 | Summarise key results with reference to study objectives | 17 |
| Limitations | 19 | Discuss limitations of the study, taking into account sources of potential bias or imprecision. Discuss both direction and magnitude of any potential bias | 19,20 |
| Interpretation | 20 | Give a cautious overall interpretation of results considering objectives, limitations, multiplicity of analyses, results from similar studies, and other relevant evidence | 17-20 |
| Generalisability | 21 | Discuss the generalisability (external validity) of the study results | 3,4, 17-21 |
| Other information | | | |
| Funding | 22 | Give the source of funding and the role of the funders for the present study and, if applicable, for the original study on which the present article is based | 23 |
| * Information is reported separately for cases and controls. | | | |

*Table S2.* Missing data of ATTRwt, ATTRwt-negative and AL discovery patients and ATTRwt and ATTRwt-negative validation patients

|  | **ATTRwt discovery (n=73)** | **AL discovery (n=55)** | **ATTRwt-negative discovery (n=59)** | **ATTRwt validation (n=35)** | **ATTRwt-negative validation (n=25)** |
| --- | --- | --- | --- | --- | --- |
| **Patient characteristics** |  |  |  |  |  |
| Age, n (%) | 0 | 0 | 0 | 0 | 0 |
| Sex, n (%) | 0 | 0 | 0 | 0 | 0 |
| HT, n (%) | 0 | 1 (2) | 0 | 0 | 0 |
| DM II, n (%) | 0 | 1 (2) | 0 | 0 | 0 |
| CAD, n (%) | 0 | 1 (2) | 0 | 0 | 0 |
| NYHA class | 0 | 0 | 0 | 0 | 0 |
| **Medication** |  |  |  |  |  |
| β-blocker, n (%) | 0 | 1 (2) | 2 (3) | 0 | 0 |
| ACEi, n (%) | 0 | 1 (2) | 2 (3) | 0 | 0 |
| ARB, n (%) | 0 | 2 (4) | 2 (3) | 0 | 0 |
| MRA, n (%) | 0 | 1 (2) | 2 (3) | 0 | 0 |
| Diuretics, n (%) | 0 | 1 (2) | 2 (3) | 0 | 0 |
| **Laboratory values** |  |  |  |  |  |
| hs-Troponin T, n (%) | 0 | 0 | 0 | 0 | 0 |
| NT-proBNP, n (%) | 1 (1) | 0 | 0 | 0 | 0 |
| eGFR, n (%) | 1 (1) | 0 | 1 (2) | 0 | 1 (4) |
| **Echocardiography** |  |  |  |  |  |
| LVEF, n (%) | 0 | 5 (9) | 1 (2) | 1 (3) | 0 |
| IVST, n (%) | 21 (28) | 11 (20) | 4 (7) | 1 (3) | 0 |
| LVMI, n (%) | 37 (50) | 13 (24) | 3 (8) | 1 (3) | 1 (4) |
| **Biomarkers discovery** |  |  |  |  |  |
| Total missing, n (%) | 0 | 0 | 93 (0.04) | - | - |
| DCN, n (%) | 0 | 0 | 0 | - | - |
| IDUA, n (%) | 0 | 0 | 0 | - | - |
| GLB-1, n (%) | 0 | 0 | 1 (2) | - | - |
| **Biomarkers validation** |  |  |  |  |  |
| DCN, n (%) | - | - | - | 0 | 0 |
| IDUA, n (%) | - | - | - | 0 | 0 |
| GLB-1, n (%) | - | - | - | 0 | 0 |
|  |  |  |  |  |  |
| Data are presented as number (%). n, number; -, not available/applicable; HT, hypertension; DMII, diabetes mellitus type 2; MI, myocardial infarction in medical history; ACEi, angiotensin converting enzyme inhibitor; ARB, angiotensin receptor blocker; MRA, mineral corticoid receptor antagonist; hs, high sensitivity; eGFR, estimated glomerular filtration rate; LVEF, left ventricular ejection fraction; IVST, intraventricular septal thickness; LVPWT, left ventricular posterior wall thickness; LVMI, left ventricular mass index; RWT, relative wall thickness. | | | | | |

*Table S3*. Serum free light chains for kappa and lambda AL-CM

|  | **κ AL-CM**  **(n=18)** | | **λ AL-CM**  **(n=37)** | |
| --- | --- | --- | --- | --- |
| κ FLC (mg/L) | 203 | [97–445] | 15 | [8–22] |
| λ FLC (mg/L) | 13 | [6–22] | 341 | [144–548] |
| κ/λ FLC ratio | 14.70 | [5.53– 40.38] | 0.06 | [0.02–0.11] |
|  |  |  |  |  |

*Table S4.* Significantly elevated and decreased biomarkers of 363 uniquely measured protein biomarkers in total, based on NPX-values of ATTRwt discovery patients (n=73) and ATTRwt-negative discovery patients (n=59)

| **Biomarker** | **Full name** | **Log2-fold change** | **Adjusted p-value** |
| --- | --- | --- | --- |
| HGF | Hepatocyte growth factor | 1.36 | 5.22×10^-8^ |
| GLB-1 | Galactosidase Beta 1 | 1.3 | 3.00×10^-8^ |
| MSLN | Mesothelin | 1.26 | 1.52×10^-7^ |
| PARP1 | Poly(ADP-ribose) polymerase 1 | 1.25 | 3.87×10^-7^ |
| FGFBP1 | Fibroblast growth factor binding protein 1 | 1.23 | 1.40×10^-8^ |
| MUC16 | Mucin 16, cell surface associated | 1.23 | 1.66×10^-5^ |
| GH1 | Growth hormone 1 | 1.13 | 4.52×10^-4^ |
| RSPO3 | R-spondin 3 | 1.06 | 5.97×10^-5^ |
| GDF2 | Growth differentiation factor 2 | 1.00 | 4.13×10^-7^ |
| S100A4 | S100 calcium binding protein A4 | 0.96 | 1.51×10^-9^ |
| DCN | Decorin | 0.92 | 1.25×10^-8^ |
| TRIM21 | Tripartite motif containing 21 | 0.9 | 2.26×10^-5^ |
| IGFBP2 | Insulin like growth factor binding protein 2 | 0.86 | 1.96×10^-6^ |
| DPP10 | Dipeptidyl peptidase like 10 | 0.85 | 8.72×10^-5^ |
| GPC1 | Glypican 1 | 0.84 | 5.55×10^-7^ |
| ANXA1 | Annexin A1 | 0.82 | 3.79×10^-6^ |
| F11R | F11 receptor | 0.80 | 3.12×10^-6^ |
| FADD | Fas associated via death domain | 0.74 | 2.51×10^-6^ |
| SLAMF7 | SLAM family member 7 | 0.73 | 3.30×10^-4^ |
| ACE2 | Angiotensin converting enzyme 2 | 0.70 | 8.15×10^-5^ |
| IL1RL1 | Interleukin 1 receptor like 1 | 0.70 | 8.31×10^-5^ |
| CXCL13 | C-X-C motif chemokine ligand 13 | 0.67 | 7.96×10^-5^ |
| ITGB2 | Integrin subunit beta 2 | 0.65 | 1.47×10^-5^ |
| EGF | Epidermal growth factor | 0.64 | 2.98×10^-3^ |
| ANPEP | Alanyl aminopeptidase, membrane | 0.63 | 8.65×10^-7^ |
| CASP3 | Caspase 3 | 0.63 | 2.19×10^-3^ |
| SPON1 | Spondin 1 | 0.63 | 8.50×10^-9^ |
| AZU1 | Azurocidin 1 | 0.62 | 3.04×10^-3^ |
| PRDX5 | Peroxiredoxin 5 | 0.62 | 1.73×10^-4^ |
| TFPI2 | Tissue factor pathway inhibitor 2 | 0.62 | 1.74×10^-6^ |
| CXADR | CXADR Ig-like cell adhesion molecule | 0.61 | 5.53×10^-4^ |
| PECAM1 | Platelet and endothelial cell adhesion molecule 1 | 0.61 | 1.86×10^-7^ |
| ICOSLG | Inducible T cell costimulator ligand | 0.59 | 1.01×10^-5^ |
| DDX58 | RNA sensor RIG-I | 0.58 | 3.83×10^-4^ |
| GP6 | Glycoprotein VI platelet | 0.57 | 8.73×10^-5^ |
| TEK | TEK receptor tyrosine kinase | 0.56 | 2.47×10^-8^ |
| TNFRSF13B | TNF receptor superfamily member 13B | 0.56 | 7.12×10^-3^ |
| SRC | SRC proto-oncogene, non-receptor tyrosine kinase | 0.55 | 3.12×10^-3^ |
| TNFRSF11B | TNF receptor superfamily member 11b | 0.55 | 3.01×10^-6^ |
| **Biomarker** | **Full name** | **Log2-fold change** | **Adjusted p-value** |
| TRIM5 | Tripartite motif containing 5 | 0.55 | 3.85×10^-3^ |
| PAPPA | Pappalysin 1 | 0.54 | 2.98×10^-5^ |
| CTSL | Cathepsin L | 0.53 | 2.02×10^-6^ |
| MPO | Myeloperoxidase | 0.53 | 3.89×10^-5^ |
| SCAMP3 | Secretory carrier membrane protein 3 | 0.53 | 4.43×10^-3^ |
| CEACAM8 | CEA cell adhesion molecule 8 | 0.52 | 2.10×10^-3^ |
| NT5E | 5'-nucleotidase ecto | 0.52 | 3.56×10^-5^ |
| OLR1 | Oxidized low density lipoprotein receptor 1 | 0.52 | 4.64×10^-4^ |
| PRTN3 | Proteinase 3 | 0.52 | 7.59×10^-3^ |
| HSD11B1 | Hydroxysteroid 11-beta dehydrogenase 1 | 0.51 | 8.81×10^-6^ |
| SELP | Selenoprotein P | 0.51 | 2.49×10^-4^ |
| AREG | Amphiregulin | 0.49 | 6.40×10^-4^ |
| TGFA | Transforming growth factor alpha | 0.49 | 6.70×10^-5^ |
| VIM | Vimentin | 0.49 | 1.09×10^-2^ |
| MMP3 | Matrix metallopeptidase 3 | 0.48 | 6.96×10^-4^ |
| ITGB1BP2 | Integrin subunit beta 1 binding protein 2 | 0.47 | 2.95×10^-3^ |
| MMP2 | Matrix metallopeptidase 2 | 0.47 | 1.61×10^-5^ |
| IL27 | Myeloid derived growth factor | 0.45 | 1.55×10^-4^ |
| CKAP4 | Cytoskeleton associated protein 4 | 0.44 | 2.74×10^-3^ |
| IGFBP7 | Insulin like growth factor binding protein 7 | 0.44 | 1.31×10^-3^ |
| CDH5 | Cadherin 5 | 0.43 | 3.14×10^-6^ |
| MMP9 | Matrix metallopeptidase 9 | 0.43 | 1.59×10^-3^ |
| GALNT3 | Polypeptide N-acetylgalactosaminyltransferase 3 | 0.42 | 4.16×10^-5^ |
| PCSK9 | Proprotein convertase subtilisin/kexin type 9 | 0.42 | 2.69×10^-4^ |
| DKK1 | Dickkopf WNT signaling pathway inhibitor 1 | 0.41 | 3.22×10^-5^ |
| NOTCH3 | Notch receptor 3 | 0.41 | 1.33×10^-5^ |
| PLAU | Plasminogen activator, urokinase | 0.41 | 2.47×10^-4^ |
| BOC | BOC cell adhesion associated, oncogene regulated | 0.40 | 1.50×10^-5^ |
| CXCL1 | C-X-C motif chemokine ligand 1 | 0.40 | 8.34×10^-3^ |
| ITGB5 | Integrin subunit beta 5 | 0.40 | 8.50×10^-7^ |
| MASP1 | MBL associated serine protease 1 | 0.40 | 6.74×10^-6^ |
| XPNPEP2 | X-prolyl aminopeptidase 2 | 0.40 | 4.01×10^-3^ |
| PRDX3 | Peroxiredoxin 3 | 0.39 | 1.41×10^-4^ |
| TFRC | Transferrin receptor | 0.39 | 4.11×10^-3^ |
| IL6 | Interleukin 6 | 0.38 | 1.87×10^-2^ |
| PDCD1LG2 | Programmed cell death 1 ligand 2 | 0.38 | 3.06×10^-5^ |
| SDC1 | Syndecan 1 | 0.38 | 2.91×10^-3^ |
| FGF2 | Fibroblast growth factor 21 | 0.37 | 9.85×10^-3^ |
| IL17RA | Interleukin 17 receptor A | 0.37 | 5.37×10^-4^ |
| ITGA6 | Integrin subunit alpha 6 | 0.37 | 1.43×10^-4^ |
| LY9 | Lymphocyte antigen 9 | 0.37 | 4.94×10^-4^ |
| CD28 | CD28 molecule | 0.36 | 9.26×10^-5^ |
|  |  |  |  |
| **Biomarker** | **Full name** | **Log2-fold change** | **Adjusted p-value** |
| AGER | Advanced glycosylation end-product specific receptor | 0.35 | 7.86×10^-4^ |
| ERBB4 | Erb-b2 receptor tyrosine kinase 4 | 0.35 | 2.83×10^-5^ |
| ICAM2 | Intercellular adhesion molecule 2 | 0.35 | 5.99×10^-5^ |
| ITGB6 | Integrin subunit beta 6 | 0.35 | 1.26×10^-4^ |
| LY75 | LY75-CD302 readthrough | 0.35 | 2.45×10^-4^ |
| WIF1 | WNT inhibitory factor 1 | 0.35 | 8.04×10^-4^ |
| ALCAM | Activated leukocyte cell adhesion molecule | 0.34 | 5.70×10^-5^ |
| BLMH | Bleomycin hydrolase | 0.34 | 2.73×10^-4^ |
| LAG3 | Lymphocyte activating 3 | 0.33 | 5.88×10^-3^ |
| MERTK | MER proto-oncogene, tyrosine kinase | 0.33 | 3.51×10^-3^ |
| AXL | AXL receptor tyrosine kinase | 0.32 | 1.63×10^-3^ |
| IGF1R | Insulin like growth factor 1 receptor | 0.32 | 1.19×10^-4^ |
| IL18 | Interleukin 18 | 0.32 | 7.28×10^-3^ |
| KPNA1 | Karyopherin subunit alpha 1 | 0.32 | 4.30×10^-3^ |
| PPP1R9B | Protein phosphatase 1 regulatory subunit 9B | 0.32 | 1.65×10^-2^ |
| HSPB1 | Heat shock protein family B (small) member 1 | 0.31 | 1.38×10^-2^ |
| IL1R1 | Interleukin 1 receptor type 1 | 0.3 | 5.48×10^-4^ |
| ITGA11 | Integrin subunit alpha 11 | 0.3 | 3.05×10^-3^ |
| PLAUR | Plasminogen activator, urokinase receptor | 0.3 | 1.42×10^-2^ |
| SORT1 | Sortilin 1 | 0.29 | 1.13×10^-4^ |
| CNTN1 | Contactin 1 | 0.28 | 3.07×10^-4^ |
| CLEC4G | C-type lectin domain family 4 member G | 0.27 | 1.13×10^-2^ |
| GRN | Granulin precursor | 0.27 | 1.26×10^-3^ |
| IL1R2 | Interleukin 1 receptor type 2 | 0.27 | 1.45×10^-3^ |
| PDGFA | Platelet derived growth factor subunit A | 0.26 | 1.65×10^-2^ |
| CD93 | CD93 molecule | 0.25 | 1.51×10^-3^ |
| FCRL3 | Fc receptor like 3 | 0.25 | 1.23×10^-2^ |
| PTX3 | Paired like homeodomain 3 | 0.25 | 1.27×10^-2^ |
| TREML2 | Triggering receptor expressed on myeloid cells like 2 | 0.25 | 1.94×10^-2^ |
| CNTNAP2 | Contactin associated protein 2 | 0.24 | 1.45×10^-2^ |
| FAS | Fatty acid synthase | 0.24 | 5.95×10^-3^ |
| SIRPA | Signal regulatory protein alpha | 0.24 | 1.94×10^-2^ |
| CD163 | CD163 molecule | 0.23 | 1.89×10^-2^ |
| ERBB2 | Erb-b2 receptor tyrosine kinase 2 | 0.23 | 1.32×10^-4^ |
| GPNMB | Glycoprotein nmb | 0.23 | 1.23×10^-6^ |
| IFNGR1 | Interferon gamma receptor 1 | 0.21 | 4.13×10^-3^ |
| ITGAV | Integrin subunit alpha V | 0.21 | 1.74×10^-5^ |
| PRELP | Proline and arginine rich end leucine rich repeat protein | 0.20 | 1.69×10^-3^ |
| ADAMTS13 | ADAM metallopeptidase with thrombospondin type 1 motif 13 | 0.12 | 7.10×10^-3^ |
| CEACAM1 | CEA cell adhesion molecule 1 | 0.11 | 5.89×10^-4^ |
| SMAD5 | SMAD family member 5 | -0.21 | 1.40×10^-3^ |
| TNFSF13 | TNF superfamily member 13 | -0.22 | 1.09×10^-2^ |
| **Biomarker** | **Full name** | **Log2-fold change** | **Adjusted p-value** |
| CXCL12 | C-X-C motif chemokine ligand 12 | -0.24 | 7.07×10^-4^ |
| MIA | MIA SH3 domain containing | -0.26 | 6.72×10^-4^ |
| NTF4 | Neurotrophin 4 | -0.29 | 6.58×10^-5^ |
| TIMP4 | TIMP metallopeptidase inhibitor 4 | -0.29 | 4.13×10^-3^ |
| TNFSF10 | TNF superfamily member 10 | -0.29 | 5.25×10^-5^ |
| LGALS1 | Galectin 1 | -0.32 | 4.53×10^-6^ |
| LGALS4 | Galectin 4 | -0.33 | 2.81×10^-3^ |
| LILRB4 | Leukocyte immunoglobulin like receptor B4 | -0.35 | 5.54×10^-3^ |
| STC1 | Stanniocalcin 1 | -0.37 | 5.33×10^-4^ |
| VSIG2 | V-set and immunoglobulin domain containing 2 | -0.40 | 1.10×10^-2^ |
| XCL1 | X-C motif chemokine ligand 1 | -0.44 | 4.46×10^-4^ |
| MEPE | Matrix extracellular phosphoglycoprotein | -0.45 | 3.56×10^-3^ |
| PTH1R | Parathyroid hormone 1 receptor | -0.50 | 5.75×10^-6^ |
| BMP-6 | Bone morphogenetic protein 6 | -0.54 | 2.21×10^-3^ |
| ACP5 | Acid phosphatase 5, tartrate resistant | -0.74 | 2.34×10^-6^ |
| KLK14 | Kallikrein related peptidase 14 | -0.77 | 1.48×10^-4^ |
| CD40LG | CD40 ligand | -0.88 | 2.74×10^-4^ |
| KLK13 | Kallikrein related peptidase 13 | -0.98 | 4.92×10^-8^ |
| FGF21 | Fibroblast growth factor 21 | -0.99 | 3.70×10^-3^ |
| ADAMTS 15 | ADAM metallopeptidase with thrombospondin type 1 motif 15 | -1.03 | 5.33×10^-9^ |
| IDUA | Alpha-L-iduronidase | -1.39 | 2.64×10^-9^ |
| LEP | Leptin | -1.63 | 4.66×10^-7^ |
| ADM | Adrenomedullin | -2.19 | 5.56×10^-6^ |
|  |  |  |  |
| All Biomarker names are according to HUGO gene nomenclature committee (HGNC) nomenclature. P-values were adjusted for multiple testing according to Benjamini-Hochberg false discovery rate method. | | | |

*Table S5****.*** Significantly elevated and decreased biomarkers in patients with AL discovery (n=55) versus ATTRwt-negative discovery (n=59) patients.

| **Biomarker** | **Log2-fold change** | **Adj. P-value** |
| --- | --- | --- |
| MSLN | 1.74 | 9.73×10^-08^ |
| PARP-1 | 1.64 | 2.12×10^-07^ |
| FGF-BP1 | 1.57 | 1.73×10^-03^ |
| HGF | 1.57 | 6.49×10^-07^ |
| GLB1 | 1.45 | 9.73×10^-08^ |
| MUC-16 | 1.41 | 9.37×10^-06^ |
| GDF-2 | 1.30 | 6.74×10^-07^ |
| S100A4 | 1.20 | 2.81×10^-08^ |
| SLAMF7 | 1.16 | 1.04×10^-05^ |
| TRIM21 | 1.12 | 6.48×10^-06^ |
| EGF | 1.10 | 9.64×10^-06^ |
| CASP-3 | 1.06 | 1.81×10^-06^ |
| SRC | 1.05 | 1.69×10^-06^ |
| GPC1 | 1.04 | 1.13×10^-06^ |
| IGFBP-2 | 1.02 | 2.17×10^-06^ |
| FADD | 0.99 | 3.84×10^-07^ |
| JAM-A | 0.99 | 5.50×10^-07^ |
| DCN | 0.94 | 9.73×10^-08^ |
| GP6 | 0.94 | 1.32×10^-06^ |
| ITGB2 | 0.91 | 2.01×10^-06^ |
| ICOSLG | 0.89 | 5.96×10^-07^ |
| SCAMP3 | 0.86 | 1.80×10^-05^ |
| GH | 0.85 | 1.69×10^-02^ |
| TRIM5 | 0.84 | 1.23×10^-05^ |
| AZU1 | 0.83 | 9.02×10^-04^ |
| DDX58 | 0.82 | 4.53×10^-05^ |
| RSPO3 | 0.81 | 8.52×10^-03^ |
| ANXA1 | 0.81 | 6.52×10^-05^ |
| TGM2 | 0.79 | 1.45×10^-04^ |
| VIM | 0.77 | 1.39×10^-03^ |
| TFPI2_mean | 0.76 | 9.14×10^-07^ |
| TNFRSF13B | 0.76 | 2.35×10^-04^ |
| PRTN3 | 0.75 | 9.41×10^-04^ |
| SELP | 0.73 | 2.24×10^-05^ |
| PECAM-1 | 0.73 | 4.30×10^-07^ |
| ST2 | 0.71 | 3.39×10^-04^ |
| TIE2 | 0.69 | 5.96×10^-07^ |
| ITGB1BP2 | 0.69 | 1.18×10^-05^ |
| ACE2 | 0.68 | 1.34×10^-04^ |
| AP-N | 0.66 | 1.52×10^-06^ |
| DPP10 | 0.66 | 3.83×10^-04^ |
| MPO | 0.65 | 1.20×10^-05^ |
| OPG | 0.64 | 1.14×10^-05^ |
| IGFBP-1 | 0.62 | 2.85×10^-02^ |
| **Biomarker** | **Log2-fold change** | **Adj. P-value** |
| DECR1 | 0.59 | 6.52×10^-05^ |
| FGF2 | 0.59 | 2.46×10^-05^ |
| PCSK9 | 0.58 | 1.23×10^-05^ |
| PRDX5 | 0.57 | 5.44×10^-03^ |
| SPON1 | 0.57 | 2.01×10^-06^ |
| SCGB3A2 | 0.56 | 4.66×10^-03^ |
| LOX-1 | 0.56 | 9.41×10^-04^ |
| ZBTB16 | 0.56 | 9.02×10^-04^ |
| CTSL1 | 0.55 | 1.32×10^-06^ |
| CXCL13 | 0.54 | 1.42×10^-03^ |
| Dkk-1 | 0.54 | 2.17×10^-06^ |
| HSD11B1 | 0.54 | 4.09×10^-05^ |
| PRDX1 | 0.54 | 8.06×10^-03^ |
| 5'-NT | 0.53 | 3.06×10^-05^ |
| PAPPA | 0.53 | 6.97×10^-05^ |
| uPA | 0.53 | 2.19×10^-05^ |
| MMP-9 | 0.51 | 1.70×10^-03^ |
| CEACAM8 | 0.50 | 4.27×10^-03^ |
| TGF-alpha | 0.50 | 3.39×10^-04^ |
| PLXNA4 | 0.50 | 9.21×10^-03^ |
| WIF-1 | 0.50 | 2.00×10^-05^ |
| STK4 | 0.50 | 6.82×10^-03^ |
| IL-17RA | 0.49 | 1.34×10^-04^ |
| HSP 27 | 0.48 | 3.64×10^-04^ |
| EDAR | 0.48 | 1.67×10^-02^ |
| EIF4G1 | 0.48 | 3.94×10^-02^ |
| IL6_mean | 0.47 | 3.18×10^-02^ |
| CXADR | 0.47 | 4.72×10^-03^ |
| NEMO | 0.47 | 9.93×10^-03^ |
| ICAM-2 | 0.45 | 6.33×10^-05^ |
| IGFBP-7 | 0.45 | 4.36×10^-03^ |
| CKAP4 | 0.44 | 3.97×10^-03^ |
| CDH5 | 0.44 | 3.47×10^-05^ |
| MILR1 | 0.43 | 9.61×10^-03^ |
| SYND1 | 0.43 | 9.41×10^-04^ |
| LY9 | 0.43 | 1.63×10^-04^ |
| XPNPEP2 | 0.42 | 6.66×10^-03^ |
| GALNT3 | 0.42 | 3.02×10^-04^ |
| PD-L2 | 0.42 | 3.95×10^-05^ |
| MMP-2 | 0.41 | 5.46×10^-04^ |
| CD28 | 0.41 | 1.53×10^-03^ |
| SORT1 | 0.41 | 1.81×10^-06^ |
| PPP1R9B | 0.41 | 3.32×10^-03^ |
| TNFRSF14 | 0.39 | 6.82×10^-03^ |
| U-PAR | 0.39 | 4.91×10^-03^ |
| BLM hydrolase | 0.39 | 5.22×10^-04^ |
| PRKCQ | 0.39 | 1.80×10^-03^ |
| **Biomarker** | **Log2-fold change** | **Adj. P-value** |
| MASP1 | 0.39 | 9.90×10^-05^ |
| MERTK | 0.38 | 3.71×10^-03^ |
| IL-27 | 0.38 | 1.13×10^-03^ |
| PDGF subunit A | 0.37 | 2.31×10^-03^ |
| ITGA6 | 0.37 | 1.65×10^-04^ |
| PON3 | 0.37 | 3.05×10^-02^ |
| IL-1RT1 | 0.37 | 7.16×10^-04^ |
| PRDX3 | 0.37 | 1.42×10^-04^ |
| IRF9 | 0.37 | 1.07×10^-02^ |
| ITGB5 | 0.36 | 8.35×10^-05^ |
| KPNA1 | 0.34 | 2.86×10^-03^ |
| BOC | 0.34 | 3.87×10^-04^ |
| TLT-2 | 0.34 | 9.21×10^-03^ |
| TRAF2 | 0.33 | 4.77×10^-02^ |
| ALCAM | 0.33 | 2.11×10^-03^ |
| IL-4RA | 0.33 | 4.18×10^-02^ |
| SHPS-1 | 0.32 | 1.79×10^-02^ |
| vWF | 0.32 | 1.00×10^-02^ |
| GRN | 0.31 | 1.10×10^-03^ |
| ERBB4 | 0.31 | 2.49×10^-04^ |
| CNTN1 | 0.31 | 2.40×10^-03^ |
| TXLNA | 0.30 | 2.12×10^-02^ |
| PAI | 0.30 | 8.36×10^-03^ |
| PADI2 | 0.30 | 3.06×10^-02^ |
| COL1A1 | 0.29 | 1.39×10^-03^ |
| AXL | 0.29 | 9.93×10^-03^ |
| AREG_mean | 0.29 | 3.94×10^-02^ |
| Notch 3 | 0.29 | 1.67×10^-02^ |
| GPNMB | 0.29 | 5.19×10^-06^ |
| ABL1 | 0.29 | 2.86×10^-02^ |
| FCRL3 | 0.28 | 1.95×10^-02^ |
| NF2 | 0.27 | 1.18×10^-02^ |
| RAGE | 0.27 | 1.38×10^-02^ |
| CD70 | 0.27 | 1.96×10^-02^ |
| ITGA11 | 0.27 | 3.05×10^-02^ |
| LTBR | 0.27 | 4.04×10^-02^ |
| CXCL16 | 0.27 | 9.32×10^-03^ |
| FAS | 0.25 | 4.07×10^-02^ |
| BIRC2 | 0.24 | 5.89×10^-03^ |
| ERBB2 | 0.24 | 7.26×10^-04^ |
| EIF5A | 0.22 | 9.17×10^-03^ |
| ICA1 | 0.22 | 1.02×10^-02^ |
| IGF1R | 0.21 | 8.73×10^-03^ |
| DAPP1 | 0.20 | 4.11×10^-02^ |
| CD48 | 0.18 | 1.04×10^-02^ |
| ITGAV | 0.17 | 7.16×10^-04^ |
| ADAM-TS13 | 0.17 | 3.85×10^-05^ |
| **Biomarker** | **Log2-fold change** | **Adj. P-value** |
| PRELP | 0.16 | 2.23×10^-02^ |
| IFN-gamma-R1 | 0.16 | 1.95×10^-02^ |
| CEACAM1 | 0.14 | 5.63×10^-05^ |
| EGFR | 0.13 | 4.37×10^-02^ |
| hOSCAR | 0.13 | 3.00×10^-02^ |
| VEGFR-3 | 0.13 | 1.39×10^-02^ |
| PIgR | -0.13 | 6.58×10^-03^ |
| MMP7 | -0.16 | 3.17×10^-02^ |
| VEGFD | -0.24 | 1.83×10^-02^ |
| PVRL4 | -0.28 | 2.85×10^-02^ |
| hK8 | -0.28 | 3.97×10^-03^ |
| CXCL12 | -0.31 | 4.97×10^-05^ |
| LPL | -0.31 | 1.58×10^-02^ |
| TRAIL | -0.32 | 8.58×10^-05^ |
| EGLN1 | -0.33 | 1.85×10^-02^ |
| TNFSF13 | -0.35 | 5.31×10^-05^ |
| SH2B3 | -0.35 | 3.94×10^-02^ |
| MAD homolog 5 | -0.35 | 9.20×10^-06^ |
| CXL17 | -0.36 | 1.02×10^-02^ |
| AGRP | -0.37 | 1.29×10^-02^ |
| KRT19 | -0.39 | 1.06×10^-02^ |
| VSIG2 | -0.39 | 2.36×10^-02^ |
| TPSAB1 | -0.39 | 1.96×10^-02^ |
| SPRY2 | -0.39 | 9.14×10^-03^ |
| IL5 | -0.39 | 4.30×10^-02^ |
| FAM3B | -0.41 | 8.09×10^-03^ |
| IL10 | -0.42 | 1.67×10^-02^ |
| CPE | -0.43 | 5.25×10^-05^ |
| CD160 | -0.43 | 2.93×10^-03^ |
| NTF4 | -0.43 | 2.00×10^-05^ |
| MEPE | -0.44 | 5.53×10^-03^ |
| Gal-1 | -0.45 | 5.50×10^-07^ |
| MIA | -0.46 | 1.81×10^-06^ |
| CCL11 | -0.49 | 4.04×10^-04^ |
| XCL1 | -0.49 | 9.41×10^-04^ |
| TNFRSF6B | -0.50 | 2.17×10^-03^ |
| LILRB4 | -0.50 | 8.14×10^-04^ |
| TIMP4 | -0.52 | 1.41×10^-04^ |
| FABP2 | -0.54 | 4.53×10^-03^ |
| Gal-4 | -0.54 | 3.97×10^-04^ |
| GIF | -0.60 | 2.83×10^-02^ |
| MMP12 | -0.65 | 3.71×10^-03^ |
| PTH1R | -0.73 | 1.42×10^-06^ |
| STC1 | -0.78 | 9.14×10^-07^ |
| BMP-6 | -0.89 | 6.34×10^-05^ |
| TR-AP | -0.98 | 1.52×10^-06^ |
| FGF-21 | -1.00 | 4.27×10^-03^ |
| **Biomarker** | **Log2-fold change** | **Adj. P-value** |
| CD40-L | -1.01 | 2.85×10^-05^ |
| KLK13 | -1.26 | 6.49×10^-07^ |
| LEP | -1.35 | 1.80×10^-05^ |
| hK14 | -1.38 | 3.84×10^-07^ |
| ADAMTS 15 | -1.44 | 1.67×10^-05^ |
| IDUA | -1.51 | 6.74×10^-07^ |
| ADM | -3.19 | 5.19×10^-06^ |
|  |  |  |
| A total of 363 unique protein biomarkers were measured by multiplex proximity extension assays. All Biomarker names are according to HUGO gene nomenclature committee (HGNC) nomenclature. The threshold for statistical significance were adjusted for multiple testing according to Benjamini-Hochberg false discovery rate method.  Adj., adjusted. | | |

*Table S6****.*** Min-max concave penalty (MCP) penalised regression results for different models, differentiating ATTRwt discovery patients (n=73), ATTRwt-negative discovery patients (n=59) and AL discovery patients (n=55) in different combinations

| **Discovery patient cohorts compared** | **Optimal**  **λ** | **Non-zero**  **coefficients** | **Cross-validation**  **error** | **R^2^** | **Signal to noise ratio** | **Prediction error** |
| --- | --- | --- | --- | --- | --- | --- |
|  |  |  |  |  |  |  |
| ATTRwt vs. ATTRwt-neg. | 0.03 | 3 | 0.14 | 0.71 | 2.43 | 0.000 |
| ATTRwt vs. AL |  | 0 |  |  |  |  |
| AL vs. ATTRwt-neg. | 0.04 | 3 | 0.15 | 0.71 | 2.44 | 0.022 |
|  |  |  |  |  |  |  |
| All MCP analyses included age, sex, a history of hypertension, diabetes mellitus, coronary artery disease as additional variables to correct for baseline characteristic differences. Test are performed according to Min-max concave penalised regression for 363 unique protein biomarkers measured by multiplex proximity extension assays. | | | | | | |

*Table S7.* Min-max concave penalty (MCP) penalised regression results for determining optimal biomarkers for the differentiation of ATTRwt discovery patients (n=73), AL discovery patients (n=55), ATTRwt-negative discovery patients (n=59) or a combination thereof.

| **Discovery patients cohorts compared** | **Adj. P-val.** | **OR** |
| --- | --- | --- |
|  |  |  |
| ATTRwt versus ATTRwt-negative |  |  |
| DCN | 3.5 × 10^-4^ | 38.6 |
| IDUA | 1.7 × 10^-3^ | 0.13 |
| GLB1 | 2.9 × 10^-2^ | 3.92 |
|  |  |  |
| ATTRwt versus AL |  |  |
| none |  |  |
|  |  |  |
| AL versus ATTRwt-negative |  |  |
| ADAMTS 15 | 5.3 × 10^-5^ | 0.13 |
| FGF-BP1 | 4.1 × 10^-4^ | 4.36 |
| DCN | 6.0 × 10^-4^ | 4.95 |
|  |  |  |
| All MCP analyses performed according to Min-max concave penalised regression (MCP) on 363 individual protein biomarkers as measured by multiplex proximity extension assays. All models included age, sex, a history of hypertension, diabetes mellitus, coronary artery disease as additional variables to correct for baseline characteristic differences. The threshold for statistical significance was adjusted for multiple testing according to Benjamini-Hochberg false discovery rate method.  Vs., versus; mFDR, method of false detection range; OR, odds ratio. | | |

*Table S8.* ATTRwt validation specific characteristics and missing variables

|  | **ATTRwt validation (n=35)** | | **Missing, n (%)** |
| --- | --- | --- | --- |
| **Patient characteristics** |  |  |  |
| CTS, n (%) | 20 | (60) | 0 |
| SCS, n (%) | 5 | (15) | 0 |
| **Laboratory values** |  |  |  |
| κ-FLC, mg/L | 25 | [21–34] | 0 |
| λ-FLC, mg/L | 18 | [16–24] | 0 |
| FLC ratio | 1.49 | ± 0.47 | 0 |
| **Fat biopsy staining** |  |  | 1 (3) |
| Congo red 0 | 18 | (53) |  |
| Congo red 1 | 11 | (32) |  |
| Congo red 2 | 3 | (9) |  |
| Congo red 3 | 1 | (3) |  |
| Congo red 4 | 1 | (3) |  |
| **Echocardiography** |  |  |  |
| GLSS, % | -11 | (-17- -8) | 3 (9) |
| **Bone scintigraphy** |  |  | 0 |
| Perugini 0 | 0 |  |  |
| Perugini 1 | 1 | (3) |  |
| Perugini 2 | 21 | (59) |  |
| Perugini 3 | 13 | (38) |  |
|  |  |  |  |
| Data are presented as mean ±SD, median [IQR], or number (%). CTS, carpal tunnel syndrome; SCS, spinal cord stenosis; NYHA, New York Health Association; FLC, free light chain. | | | |

*Table S9.* Comparison of ATTRwt discovery patients with ATTRwt validation patients

|  | **ATTRwt discovery (n=73)** | | **ATTRwt validation (n=35)** | |  | **p-value** | |
| --- | --- | --- | --- | --- | --- | --- | --- |
| **Patient characteristics** |  |  |  |  |  |  |  |
| Age, years | 74 | ± 7 | 77 | ± 6 |  |  | 0.05 |
| Men, n (%) | 67 | (92) | 30 | (86) |  |  | 0.33 |
| HT, n (%) | 11 | (15) | 15 | (43) |  |  | **0.002** |
| DM II, n (%) | 7 | (10) | 6 | (17) |  |  | 0.26 |
| CAD, n (%) | 3 | (4) | 3 | (9) |  |  | 0.34 |
| **Functionality** |  |  |  |  |  |  |  |
| NYHA class, n (%) |  |  |  |  |  |  | 0.37 |
| *I* | 9 | (12) | 2 | (6) |  |  |  |
| *II* | 42 | (58) | 24 | (69) |  |  |  |
| *III* | 22 | (30) | 8 | (23) |  |  |  |
| *IV* | 0 |  | 1 | (3) |  |  |  |
| **Medication** |  |  |  |  |  |  |  |
| β-blocker, n (%) | 47 | (64) | 21 | (60) |  |  | 0.40 |
| ACEi, n (%) | 19 | (26) | 14 | (40) |  |  | 0.14 |
| ARB, n (%) | 6 | (8) | 9 | (26) |  |  | **0.01** |
| MRA, n (%) | 38 | (52) | 15 | (43) |  |  | 0.37 |
| Diuretics, n (%) | 68 | (93) | 19 | (54) |  | **<** | **0.001** |
| **Prognostic** |  |  |  |  |  |  |  |
| NAC stage |  |  |  |  |  |  | 0.12 |
| *Stage 1* | 29 | (40) | 18 | (51) |  |  |  |
| *Stage 2* | 31 | (43) | 15 | (43) |  |  |  |
| *Stage 3* | 12 | (17) | 2 | (6) |  |  |  |
| **Laboratory values** |  |  |  |  |  |  |  |
| hs-Troponin T, ng/L | 55 | [40–75] | 46 | [32–57] |  |  | **0.04** |
| NT-proBNP, ng/L | 3127 | [1826–5239] | 2044 | [1201–3434] |  |  | 0.21 |
| eGFR, ml/min 1.73m^2^ | 59 | ± 16 | 62 | ± 18 |  |  | 0.40 |
| **Echocardiography** |  |  |  |  |  |  |  |
| LVEF, % | 46 | ± 10 | 51 | ± 10 |  |  | **0.02** |
| IVST, mm | 18 | ± 3 | 17 | ± 3 |  |  | 0.19 |
| LVMI, g/m^2^ | 176 | ± 52 | 166 | ± 59 |  |  | 0.47 |
|  |  |  |  |  |  |  |  |
| Data are presented as mean ±SD, median [IQR], or number (%). Significance was calculated following an unpaired t-test for normally distributed continuous variables, Mann-Whitney U test for skewed continuous variables and χ^2^ test for categorical variables. HT, hypertension; DMII, diabetes mellitus type 2; CAD, coronary artery disease in medical history; ACEi, angiotensin converting enzyme inhibitor; ARB, angiotensin receptor blocker; MRA, mineral corticoid receptor antagonist; Diuretics consist of loop- and thiazide diuretics; NAC, national amyloidosis centre stage, based on eGFR and NT-proBNP; eGFR, estimated glomerular filtration rate; hs, high sensitivity; LVEF, left ventricular ejection fraction; IVST, interventricular septal thickness at diastole; LVMI, left ventricular mass index. | | | | | | | |

*Table S10.* Comparison of ATTRwt-negative discovery patients with ATTRwt-negative validation patients

|  | **ATTRwt-negative discovery (n=59)** | | **ATTRwt-negative validation**  **(n=25)** | | |
| --- | --- | --- | --- | --- | --- |
| **Patient characteristics** |  |  |  |  |  |
| Age, years | 72 | ± 7 | 74 | ± 10 |  |
| Men, n (%) | 31 | (53) | 15 | (60) |  |
| HT, n (%) | 47 | (80) | 21 | (84) |  |
| DM II, n (%) | 21 | (36) | 12 | (48) |  |
| CAD, n (%) | 14 | (24) | 4 | (16) |  |
| **Functionality** |  |  |  |  |  |
| NYHA class, n (%) |  |  |  |  |  |
| I | 0 |  | 0 |  |  |
| II | 38 | (64) | 14 | (56) |  |
| III | 21 | (36) | 11 | (44) |  |
| IV | 0 |  | 0 |  |  |
| **Medication** |  |  |  |  |  |
| β-blocker, n (%) | 49 | (83) | 18 | (82) |  |
| ACEi, n (%) | 18 | (31) | 5 | (23) |  |
| ARB, n (%) | 13 | (22) | 4 | (18) |  |
| MRA, n (%) | 21 | (42) | 10 | (46) |  |
| Diuretics, n (%) | 50 | (86) | 20 | (91) |  |
| **Laboratory values** |  |  |  |  |  |
| hs-Troponin T, ng/L | 20 | [14–35] | 28 | [24–49] |  |
| NT-proBNP, ng/L | 1569 | [740–2966] | 1665 | [948–3848] |  |
| eGFR, ml/min/1.73m^2^ | 56 | ± 23 | 45 | ± 16 |  |
| **Echocardiography** |  |  |  |  |  |
| LVEF, % | 54 | ± 7 | 52 | ± 6 |  |
| IVST, mm | 11 | ± 2 | 12 | ± 3 |  |
| LVMI, g/m^2^ | 100 | ± 31 | 101 | ± 30 |  |
|  |  |  |  |  |  |
| Data are presented as mean ±SD, median [IQR], or number (%). No significance of difference was calculated due to randomised sample selection.  n, number; HT, hypertension; DMII, diabetes mellitus type 2; CAD, coronary artery disease in medical history; ACEi, angiotensin converting enzyme inhibitor; ARB, angiotensin receptor blocker; MRA, mineral corticoid receptor antagonist; Diuretics consist of loop- and thiazide diuretics; eGFR, estimated glomerular filtration rate; LVEF, left ventricular ejection fraction; IVST, interventricular septal thickness at end diastole; LVMI, left ventricular mass index. | | | | | |

*Table S11.* Predictive values for differentiating ATTRwt validation (n=35) and ATTRwt-negative validation (n=25) patients.

|  | **DCN** | |  | **IDUA** | | | |
| --- | --- | --- | --- | --- | --- | --- | --- |
| **Cut-off** | ≥ 2895 | |  | ≤ 3.61 | | ≤ 2.17 | |
| **TP, n** | 32 | |  | 32 | | 21 | |
| **FP, n** | 12 | |  | 9 | | 3 | |
| **FN, n** | 3 | |  | 3 | | 14 | |
| **TN, n** | 13 | |  | 16 | | 22 | |
| **Sensitivity, %** | 91 | |  | 91 | | 60 | |
| **Specificity, %** | 52 | |  | 64 | | 88 | |
| **LR+** | 1.94 | |  | 2.54 | | 5.00 | |
| **LR-** | 0.16 | |  | 0.13 | | 0.45 | |
| **Accuracy, %** | 75 | |  | 80 | | 72 | |
| **Prevalence, %** | 58 | 10 |  | 58 | 10 | 58 | 10 |
| **PPV, %** | 73 | 17 |  | 78 | 22 | 88 | 36 |
| **NPV, %** | 81 | 98 |  | 84 | 99 | 61 | 95 |
| **NNS, n** | 1.4 | 5.8 |  | 1.7 | 4.5 | 1.1 | 2.8 |
|  |  |  |  |  |  |  |  |
| n, number; TP, true positive; FP, false positive; FN, false negative; TN, true negative; LR, likelihood ratio; PPV, positive predictive value; NPV, negative predictive value; NNS, number needed to scan | | | | | | | |

*Table S12.*  Association of DCN and IDUA with conventional disease markers

|  | **DCN (per pg/mL increment)** | | | | | | |
| --- | --- | --- | --- | --- | --- | --- | --- |
|  | **ATTRwt validation**  **(n=35)** | | |  | **ATTRwt-negative validation (n=25)** | | |
|  | **R^2^** | **β** | **Sign.** |  | **R^2^** | **β** | **Sign.** |
| **Patient characteristics** |  |  |  |  |  |  |  |
| Age, years | 0.02 | 32 |  |  | 0.11 | -29 |  |
| **Laboratory values** |  |  |  |  |  |  |  |
| hs-Troponin, ng/mL | 0.01 | 0.01 |  |  | 0.05 | -0.01 |  |
| NT-proBNP, pg/mL | 0.04 | 0.08 |  |  | 0.08 | 0.01 |  |
| eGFR, ml/min/1.73m^2^ | 0.04 | 10 |  |  | 0.03 | 13 |  |
| IDUA, ng/mL | 0.01 | -162 |  |  | 0.01 | 17 |  |
| **TTE** |  |  |  |  |  |  |  |
| LVEF, % | 0.01 | -14 |  |  | 0.08 | -43 |  |
| IVST, mm | 0.08 | -43 |  |  | 0.01 | -9 |  |
| LVMI, g/m^2^ | 0.01 | -1 |  |  | 0.03 | -4 |  |
|  |  |  |  |  |  |  |  |
|  | **IDUA (per ng/mL increment)** | | | | | | |
|  | **ATTRwt validation**  **(n=35)** | | |  | **ATTRwt-negative validation (n=25)** | | |
|  | **R^2^** | **β** | **Sign.** |  | **R^2^** | **β** | **Sign.** |
| **Patient characteristics** |  |  |  |  |  |  |  |
| Age, years | 0.001 | 0.02 |  |  | 0.12 | -0.06 |  |
| **Laboratory values** |  |  |  |  |  |  |  |
| hs-Troponin, ng/mL | 0.03 | -0.63 |  |  | 0.01 | -2.00 |  |
| NT-proBNP, pg/mL | 0.04 | -0.001 |  |  | 0.02 | -0.001 |  |
| eGFR, ml/min/1.73m^2^ | 0.03 | 0.02 |  |  | 0.01 | 0.03 |  |
| **TTE** |  |  |  |  |  |  |  |
| LVEF, % | 0.03 | 0.04 |  |  | 0.04 | 0.05 |  |
| IVST, mm | 0.001 | -0.03 |  |  | 0.01 | -0.16 |  |
| LVMI, g/m^2^ | 0.02 | -0.01 |  |  | 0.03 | -0.01 |  |
|  |  |  |  |  |  |  |  |
| Significance (Sign.) is calculated in linear regression: empty represents a p-value >0.05, * ≤0.05, ** ≤0.01, *** ≤0.001. hs, high sensitivity; eGFR, estimated glomerular filtration rate; LVEF, left ventricular ejection fraction; IVST, interventricular septal thickness at end diastole; LVMI, left ventricular mass index. | | | | | | | |
